# LytF contributes to pilus extrusion during natural competence in *Streptococcus sanguinis* SK36

**DOI:** 10.1128/jb.00118-26

**Published:** 2026-04-22

**Authors:** Rebekka Moe, Katarzyna Wiaroslawa Piechowiak, Leiv Sigve Håvarstein, Morten Kjos, Daniel Straume

**Affiliations:** 1 Faculty of Chemistry, Biotechnology and Food Science, Norwegian University of Life Sciences 56625, Ås, Norway; University of Notre Dame, Notre Dame, Indiana, USA

**Keywords:** LytF, *Streptococcus sanguinis*, type IV pilus, natural transformation

## Abstract

Streptococci may enter a physiological state called competence, during which they express a specific set of genes required for exogenous DNA uptake and its subsequent integration into the genome through homologous recombination. This process, termed natural transformation, facilitates the horizontal acquisition of genetic material, potentially conferring adaptive advantages that enhance bacterial survival under selective pressures. To make homologous DNA available in the surrounding environment, *Streptococcus pneumoniae* expresses a cell wall hydrolase (CbpD) that lyses and kills closely related species. This process has been coined *fratricide*, and the acting hydrolase a *fratricin*. A significant fraction of streptococcal species does not encode a CbpD-like protein, but another competence-induced peptidoglycan hydrolase LytF. It has been speculated that LytF serves the same purpose as CbpD; however, our investigations into the role of LytF in *Streptococcus sanguinis* revealed no evidence supporting LytF as a fratricin. Instead, we show that LytF is involved in natural transformation by promoting DNA uptake. An essential part of DNA uptake is the competence-induced type IV pilus, which facilitates DNA uptake by pulling nearby DNA toward the cell. By immunoblotting and microscopy imaging, we found that LytF increases extracellular levels of the major pilus component ComGC, suggesting that LytF may modify peptidoglycan to promote pilus extrusion across the cell wall, thereby enhancing the efficiency of DNA uptake.

**IMPORTANCE:**

Streptococci are a significant cause of severe infections in both humans and animals. They are particularly adept at acquiring new genes through horizontal gene transfer, as they can become competent for natural transformation. This allows them to quickly adapt to selective pressure and spread genes involved in virulence and antibiotic resistance. In *Streptococcus sanguinis*, the competence-induced peptidoglycan hydrolase LytF has been reported to stimulate natural transformation. Our study contributes to understanding this process by demonstrating that LytF promotes extrusion of the transformation pilus required for DNA uptake.

## INTRODUCTION

Most species of the genus *Streptococcus* appear to have the genes required to enter a state in which they become competent for natural genetic transformation (1). During the competent state, streptococci can take up naked DNA from the environment and incorporate it into the genome by homologous recombination. The competent state is regulated by the extracellular concentration of specific peptide pheromones (2, 3). Two different pheromone-based systems are found to regulate entrance to competence in streptococcal species. The ComCDE system, which was first discovered in *Streptococcus pneumoniae* (2, 4, 5), is used by members of the mitis and anginosus phylogenetic groups, including *Streptococcus sanguinis*, while members of the bovis, mutans, salivarius, and pyogenic groups use the ComRS system (6, 7). Streptococci expressing the ComCDE system respond to the extracellular concentration of CSP (competence-stimulating peptide encoded by *comC*), whereas species expressing the ComRS system are induced by XIP (*comX*-inducing peptide encoded by *comS*). A large diversity of these two pheromones is found in nature, ensuring species and even strain-specific signaling (6, 8, 9). When a certain level of extracellular peptide pheromone is reached, it triggers a signaling cascade, which leads to the expression of genes necessary for entering the competent state. The genes transcribed during the competent state are divided into two main categories: the early and late genes. Among others, gene products involved in CSP/XIP production, transport, and processing are considered to be early competence genes. The transition from early to late stages of competence is typically defined by the transcription of *comX*, encoding an alternative sigma factor that induces transcription of the late genes (10, 11). These are genes associated with DNA uptake and homologous recombination (for review, see reference [1]). In addition, most streptococcal species have a late competence gene encoding a secreted, peptidoglycan hydrolase. There are two main types of these competence-specific peptidoglycan hydrolases: those related to CbpD and those belonging to the LytF group (12).

CbpD was first described in *S. pneumoniae* (13, 14). This enzyme is secreted by competent pneumococci and has been shown to attack and lyse non-competent pneumococci as well as members of the closely related species *Streptococcus mitis* and *Streptococcus oralis* (15). Because CbpD only kills susceptible sibling cells and close relatives of *S. pneumoniae*, this predatory mechanism has been termed fratricide, and the competence-induced murein hydrolase a fratricin. The fratricide mechanism is proposed as a strategy for the competent cell population to gain access to homologous DNA, which can be integrated into the genomes, thereby improving the chances of obtaining beneficial traits in response to environmental challenges (15). To avoid committing suicide by self-produced CbpD, competent pneumococci express an early competence gene encoding an integral membrane protein termed ComM that protects against CbpD (16). Evidence suggests that ComM works together with LytR (LytR-CpsA-Psr family protein) to alter the ratio of lipoteichoic to wall teichoic acids (17). In addition, overexpression of ComM results in cell elongation, and it has recently been shown to delay septal peptidoglycan synthesis (18–20). However, the exact mechanism of ComM-mediated immunity remains unclear. The pneumococcal CbpD consists of an N-terminal cysteine/histidine-dependent amidohydrolase/peptidase (CHAP) domain, one or two Src homology 3b (SH3b) domains, and a C-terminal choline-binding domain (Cbd) consisting of four choline-binding motifs (21, 22). The Cbd module mediates binding of CbpD to choline residues decorating pneumococcal lipoteichoic acid (LTA) and wall teichoic acid (WTA), while the SH3b domain likely binds to the peptidoglycan part of the cell wall (23). It has been shown that CbpD specifically cleaves nascent peptidoglycan synthesized by the divisome and consequently splits the cell wall of susceptible streptococci along the cell equator (24).

As mentioned above, many streptococcal species do not express CbpD-like fratricins during competence. Instead, they express an unrelated peptidoglycan hydrolase termed LytF. Species carrying the *lytF* gene include several members of the mitis and mutans phylogenetic groups, as well as all members of the anginosus and bovis groups. LytF proteins have 3–5 Bsp (group B streptococcal secreted protein) repeats at their N-termini and a CHAP domain at their C-terminal end (25). Muralytic activity has been demonstrated for LytF in *Streptococcus gordonii* and *S. sanguinis* using zymography, and it has been shown to cause the release of DNA from susceptible oral streptococci (25–29). Localization studies of LytF in *S. gordonii* showed that the Bsp repeats bind to the septal regions and cell poles, resembling the binding pattern of CbpD (25). Furthermore, in *S. sanguinis,* the early competence gene *SSA_0195* (new locus tag: *SSA_RS01125*) encodes a protein with weak homology (23.5% identity) to *S. pneumoniae* CbpD-immunity protein ComM (30). These results indicate that LytF may be a functional analog to CbpD, but to the best of our knowledge, a fratricide mechanism involving LytF as a lytic enzyme has yet to be described.

In some streptococcal species, mutants lacking LytF or a CbpD-like protein show reduced transformation efficiency when provided similar concentrations of purified DNA, that is, no fratricide is required to release homologous DNA from target cells (29, 31–33). Compared to their parental strains, these mutants exhibit reduced transformation rates ranging from 4-fold (*S. sanguinis*) to more than 33-fold (*S. mutans*) (29, 32). This puzzling observation has led to speculations that these murein hydrolases may not be involved in fratricide but have a different, or additional, role during competence that influences DNA uptake or recombination (31). An essential structure for DNA uptake is a DNA-binding type IV pilus, which extends from the cell surface to bring the transforming DNA into the DNA uptake machinery. It is encoded by the *comG* operon and, consisting of only five pilins and two assembly proteins, it is the simplest type IV pilus yet discovered. The filament is mainly made up of ComGC major pilin units, while the four minor pilins ComGD-GG form a complex making up the DNA-binding pilus tip complex (34, 35). Protrusion of the pilus is thought to be powered by the ATPase ComGA docked by a multimer of ComGB constituting the assembly platform (36, 37). Interestingly, the width of the pilus-DNA complex (7–8 nm) is wider than the pore sizes estimated for Gram-positive peptidoglycan (6 nm), suggesting that the cell wall acts as a physical barrier that the pilus needs to penetrate to allow extension into the extracellular milieu (37, 38). How this is accomplished by the competent cells is not understood, but it has been hypothesized that specific cell wall remodeling enzymes are required to make space for the transformation pilus, similar to the role such enzymes play in assembling macromolecular structures in Gram-negative bacteria (39–41).

In the present study, we have investigated the function of LytF in *S. sanguinis* SK36. Our results did not support a role of LytF as a fratricin analogous to the pneumococcal CbpD. Instead, by using non-recombining DNA and immunodetection of the pilus, we show that the reduced transformation efficiency of a Δ*lytF* mutant is caused by significantly lower extracellular levels of the competence pilus, strongly suggesting that LytF is important for the extrusion of the transformation pilus across the cell wall in *S. sanguinis*.

## RESULTS

### Exploring the potential role of LytF in fratricide

**Fig 1:**
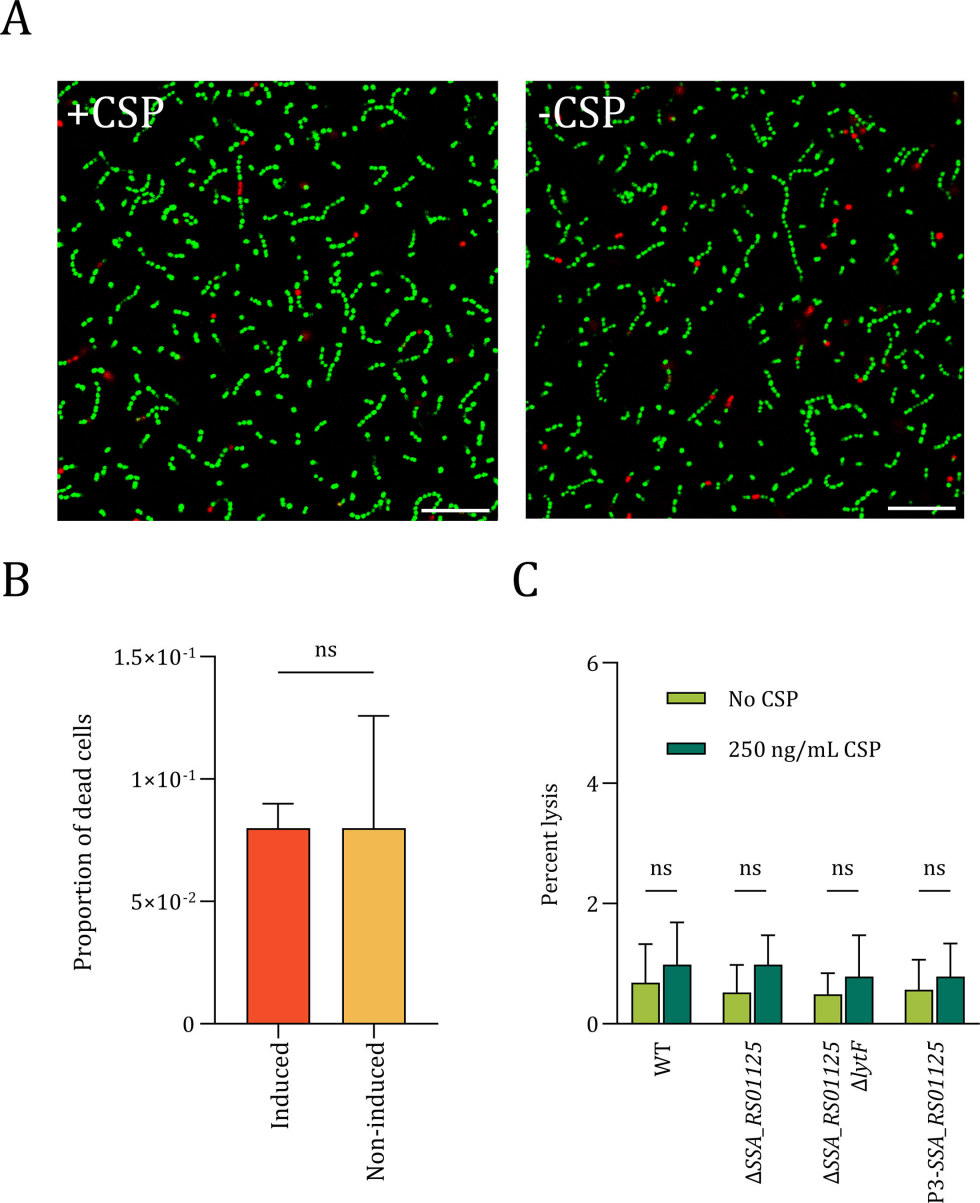
Self-lysis assay of competent Δ*SSA_RS01125* cells. *S. sanguinis* SK36 cells stained using the BacLight Live/Dead kit, which stains live bacteria green and dead bacteria red. Panel **A**: Live/dead staining of induced (+CSP) and non-induced (−CSP) cells of KP55 (Δ*comC*, Δ*SSA_RS01125*). Scale bars are 10 µm. Panel **B**: Proportion of dead cells in the induced and non-induced cultures. The difference between the means was not statistically significant, as determined by a Student’s *t*-test (*P* > 0.05). A total of 2,348 cells from three technical replicates were counted. (**C**) Comparison of autolysis in competence-induced *SSA_RS01125^+^* and *SSA_RS01125*^‒^ cells. The following mutant strains were tested: DS921(Δ*comC*, *lacZ*^+^), DS922 (Δ*comC*, Δ*SSA_RS01125*, *lacZ^+^*), DS934 (Δ*comC*, Δ*SSA_RS01125*, Δ*lytF*, *lacZ*^+^), and DS926 (Δ*comC*, P3-*SSA_RS01125*, *lacZ*^+^). In the latter strain, DS926, expression of SSA_RS01125 is driven by a constitutive synthetic promoter. The four mutants were induced to competence at OD_550_ = 0.3 and grown for another 60 minutes. Non-induced cultures served as negative controls. The relative levels of extracellular β-galactosidase activity suggested that no significant LytF-mediated autolysis occurred in any of the strains during competence. Results are based on data from three biologically independent experiments. The difference between the means was not statistically significant for any strain, as determined by a Student’s *t*-test (*P* > 0.05).

It has been hypothesized that the murein hydrolase LytF is involved in a fratricide mechanism in streptococci, analogous to what has been demonstrated for the murein hydrolase CbpD in *S. pneumoniae*. This is based on the observation that non-competent *S. sanguinis* SK36 treated with LytF results in a growth medium containing more extracellular DNA compared to non-treated cultures, indicating that LytF-mediated cell lysis had taken place (29). If LytF takes part in a fratricide mechanism, it is reasonable to believe that *S. sanguinis* expresses a LytF-immunity protein analogous to the CbpD-immunity protein ComM found in *S. pneumoniae*. In *S. pneumoniae*, deletion of *comM* results in 20%–30% self-lysis of the competent population and a significant drop in the culture’s optical density (OD) due to production of CbpD (16, 42). No LytF immunity protein has been identified; however, *S. sanguinis* SK36 encodes a competence-induced ComM-like protein (*SSA_RS01125*) expressed during the early stage of competence (30). We therefore explored whether competence induction could result in self-lysis in Δ*SSA_RS01125* cells, due to exposure to self-produced LytF. Unexpectedly, no decline in OD was observed after induction of competence by the addition of exogenous CSP in the Δ*SSA_RS01125* mutant (KP55), suggesting that LytF secreted by the competent KP55 cells did not cause autolysis observable by OD measurements (Fig. S1). To further explore the lytic potential of LytF, we performed both live/dead staining and β-galactosidase release assays to detect self-lysis of competent Δ*SSA_RS01125* cells (Fig. 1). The competence-induced Δ*SSA_RS01125* cultures did, however, not show a significant increase in the number of dead cells nor increased release of β-galactosidase, suggesting that LytF is not able to cause self-lysis of Δ*SSA_RS01125* cells (Fig. 1).

**Fig 2:**
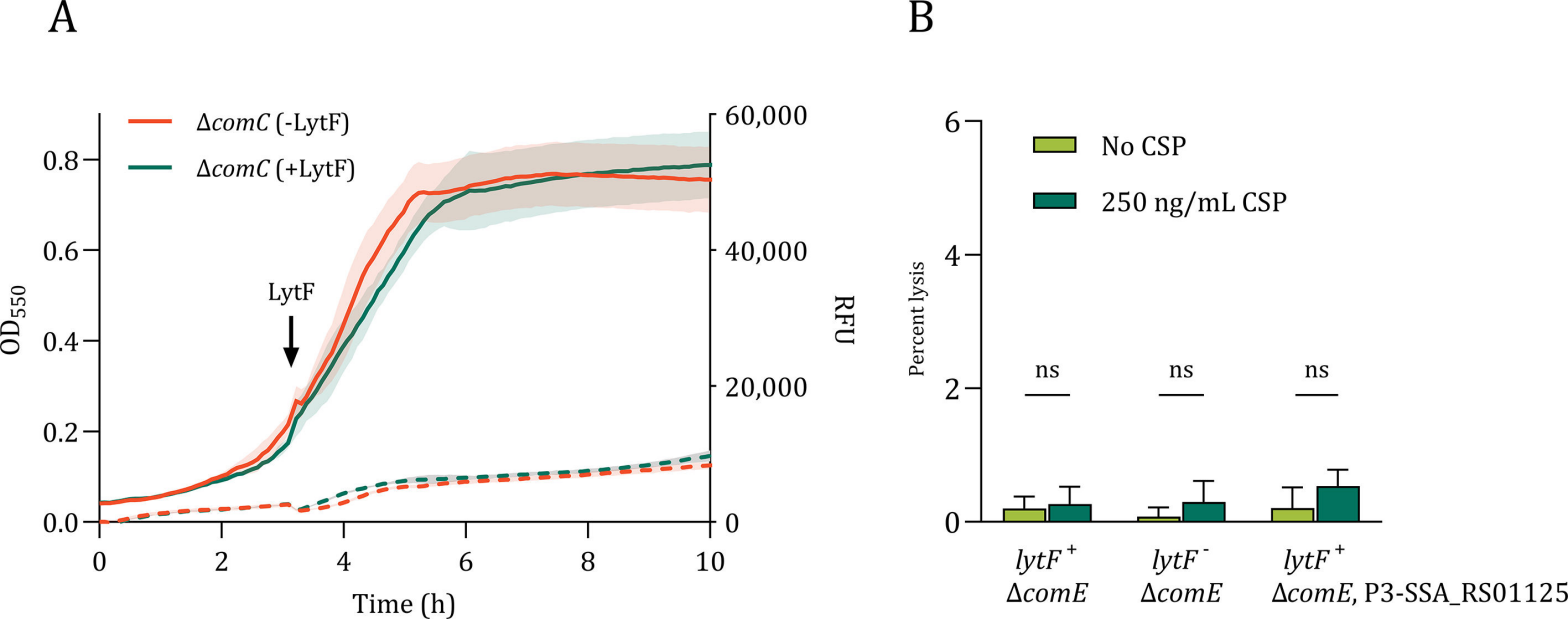
Lytic activity of LytF on non-competent *S. sanguinis* SK36. (**A**) LytF lysis assay using SYTOX. Purified His_6_-LytF was added to a final concentration of 5 µg/mL to a Δ*comC* (KP52) culture at OD_550_ = 0.2. The same volume of PBS was added to a parallel culture as a negative control. The addition of His_6_-LytF did not increase the relative fluorescent signal nor cause a drop in OD_550_, meaning no lysis was detected. (**B**) Percent intracellular β-galactosidase released from non-competent *S. sanguinis* SK36 (Δ*comE*) when co-cultivated with LytF proficient and deficient attacker cells. P3-*SSA_RS01125* cells express SSA_RS01125 constitutively. Attacker cells expressing LytF did not significantly increase the levels of β-galactosidase found in the culture supernatants. The difference between the means was not statistically significant for any strain, as determined by a Student’s *t*-test (*P* > 0.05). Both experiments were performed using at least three biological replicates.

Since no self-lysis was observed, we wondered whether competent *S. sanguinis* cells become immune to LytF by expressing a different factor than SSA_RS01125. We tested this possibility (i) by measuring cell lysis of non-competent cells treated with purified LytF and (ii) by performing a co-cultivation experiment involving competent attacker cells (LytF producers) and non-competent target cells (Δ*comE* cells) (Fig. 2). To obtain purified LytF, we replaced the native *lytF* with a His_6_ version in *S. sanguinis* and purified His_6_-LytF from the supernatant of a competent culture (Fig. S2). His_6_-LytF was then added to exponentially growing Δ*comC* (KP52) cultures at OD_550_ = 0.2. This *comC* knockout mutant is unable to auto-induce competence and, therefore, cannot express any competence-induced immunity factor. Like for the self-lysis experiment, the addition of 5 µg/mL His_6_-LytF did not result in cell lysis, as determined by the absence of any decrease in OD_550_ and the lack of fluorescence (SYTOX Green) suggesting that no DNA was released (Fig. 2A). Lytic activity of the purified His_6_-LytF enzyme used in this assay was confirmed by zymography and the amount added exceeded that found produced in competent cells of a 350 µL culture at OD_550_ = 0.2 (Fig. S3).

Next, we tested whether intracellularly expressed β-galactosidase was released from non-competent *S. sanguinis* SK36 target cells when grown together with LytF-producing attacker cells. This technique has been successfully used to measure fratricin-mediated lysis in *S. pneumoniae* and *S. thermophilus* (15, 31, 42). The *lacZ* gene from *Escherichia coli* was inserted into the middle of a gene of unknown function (*SSA_RS09615*) behind a constitutive extended −10 promoter (see methods) in a *S. sanguinis* Δ*comE* background (unable to induce competence). We set up a series of experiments in which a competence-induced attacker strain secreting LytF was co-cultivated with a competence-deficient (Δ*comE*) target strain. The following pairs of attacker and target strains were tested: KP52 (Δ*comC*) + DS937 (Δ*comE*, *lacZ^+^*), KP61 (Δ*comC*, Δ*lytF*) + DS937, and KP52 + DS939 (Δ*comE*, P3-*SSA_RS01125*, *lacZ*^+^). However, no significant LytF-mediated release of β-galactosidase was detected in any of the co-cultivation experiments (Fig. 2B). Hence, although LytF has been shown to be a peptidoglycan hydrolase in zymogram assays (29), we could not detect any signs of LytF-induced lysis in competent Δ*SSA_RS01125* or non-competent cells, showing that its lytic activity is limited under the laboratory conditions used here.

### LytF improves DNA uptake in competent *S. sanguinis*

In pneumococci, the fratricide key enzyme (CbpD) has been shown to drastically increase the rate of horizontal gene transfer in a co-culture (15), whereas it does not seem to be important for transformation rates when competent cells are provided naked homologous DNA (Fig. S4). For *S. sanguinis*, *S. mutans*, *S. thermophilus*, and *S. suis*, on the other hand, their putative fratricins are reported to increase transformation rates when naked DNA is available (29, 31–33). Since the cells are given equal amounts of homologous DNA, one would reason that it should make the fratricins obsolete in transformation, suggesting that the competence-induced murein hydrolases in these species play a role in natural transformation that does not involve lysis of target cells. To investigate how LytF affects the transformation rate, we conducted a series of transformation efficiency assays in relevant mutant strains.

**Fig 3:**
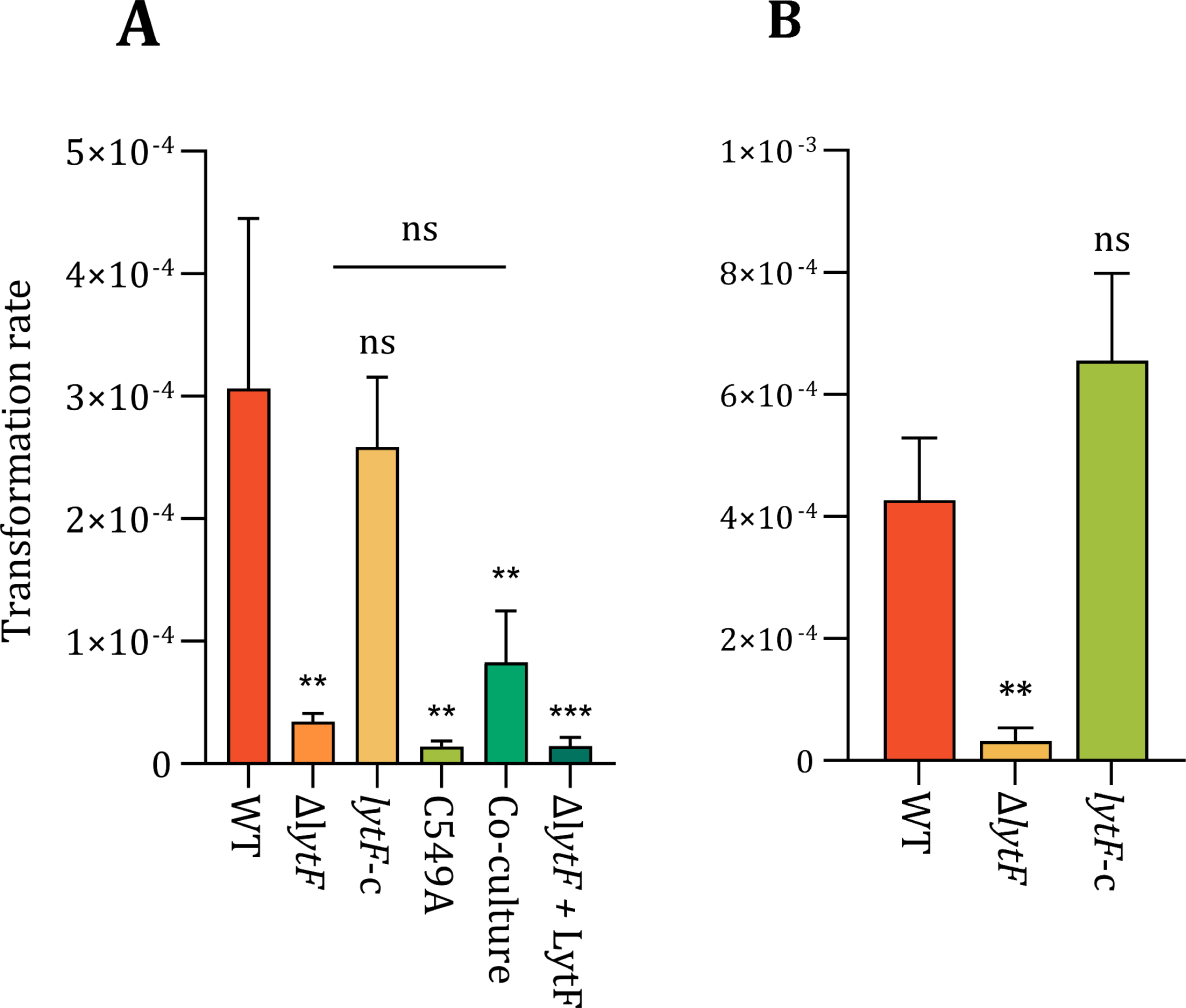
Reduced transformation in *lytF*-deficient cells. Panel **A**: Cultures were induced to competence at OD_550_ = 0.2, supplied with 250 ng/mL linear transforming DNA (Δ*SSA_RS01125::janus*). The Δ*lytF* mutant (KP61) transformed on average nine times less efficiently than the wild-type strain (KP52). The transformation rate was restored to wild-type levels in the complementation strain (RM125) in which *lytF* has been ectopically reintroduced into the genome (*lytF-c*). The addition of LytF extracellularly, either by co-cultivation of a *lytF*^-^ strain (KP54) with a *lytF*^+^ strain (KP52) or by supplementing a *lytF*^-^ strain (KP61) with purified His_6_-LytF (+ LytF), did not significantly improve transformation rates. In the co-cultivation experiment, a Δ*SSA_RS01125::aad9* cassette was used as the transforming DNA. A strain (RM133) in which the catalytic cysteine in the active site of LytF was substituted for an alanine (C549A) exhibited a transformation rate similar to that of the Δ*lytF* strains. Statistical significance was determined using a one-way ANOVA and Dunnett test, * = *P* ≤ 0.05, ** = *P* ≤ 0.01, ns = *P* > 0.05. Panel **B**: Same as above, except that the cultures were supplied with transforming DNA in the form of a plasmid (pFD116). In these experiments, the Δ*lytF* mutant transformed on average 11 times less efficiently. Student’s t-test, * = *P* < 0.05. All experiments were performed using at least three biological replicates.

Since transformation efficiency may vary with cell density, we first optimized the transformation protocol by comparing the transformation efficiencies of the wild type and the Δ*lytF* mutant when induced with CSP at different stages of the exponential growth phase (Table S3). The highest transformation efficiencies were obtained with cultures at OD_550_ = 0.2–0.4, resulting in a transformation rate of 0.036%–0.054%. Transformation was nearly abolished when inducing at OD_550_ > 0.6. In light of this observation, we performed all transformation experiments at OD_550_ = 0.2. The transformation rate of the wild-type strain observed in these experiments was lower than previous reports for *S. sanguinis* (29, 34), possibly due to variations in experimental conditions, the choice of genetic markers, target loci, or strains. We ruled out that this distinction was caused by insufficient competence induction by measuring the fraction of competent cells after CSP induction using fluorescence microscopy of strains in which a gene encoding sfGFP was placed just downstream of *comX* (Fig. S4). In the induced cultures, ~80% of the cells were competent, which is in line with what has been reported for other streptococcal species (43, 44). When transforming the KP52 (Δ*comC*) and KP61 (Δ*comC*, Δ*lytF*) strains using a linear DNA Δ*SSA_RS01125::janus* cassette, the Δ*lytF* mutant displayed an average reduction in the number of transformants by approximately ninefold, which is comparable with the results of Cullin et al. (Fig. 3A). The reintroduction of *lytF* with its native promoter in a neutral site (*SSA_RS05615*) in the KP61 genome restored the transformation efficiency to wild-type levels. A strain in which the cysteine (C549) in the active site of the LytF CHAP domain was substituted for an alanine (RM133) transformed with levels similar to the Δ*lytF* mutant, suggesting that the cells require the muralytic activity of LytF for natural transformation to be efficient.

LytF has a predicted signal sequence for secretion and retains zymogram activity after secretion (29). To determine whether the activity of LytF in promoting natural transformation is also maintained extracellularly, we first co-cultured the Δ*lytF*-mutant KP54 (Δ*comC*, Δ*lytF::janus*) with the LytF-producing wild-type strain KP52. Co-cultivation did, however, not significantly increase the transformation rate of the mutant (Fig. 3A). In addition, we supplemented the Δ*lytF* mutant strain KP61 with purified His_6_-LytF (5 µg/mL) and, similar to the co-culture results, the addition of purified LytF failed to restore the transformation efficiency to wild-type levels (Fig. 3A). This suggests that secreted LytF does not improve transformation rates in strains other than its native producer.

Since natural transformation depends on both DNA uptake and homologous recombination, a disruption in either process could account for the reduced transformation rate seen in fratricin mutants. We hypothesized that the reduced transformation efficiency of the *S. sanguinis* SK36 Δ*lytF* mutant was linked to reduced DNA uptake. To rule out an effect on homologous recombination, we compared transformation rates using linear recombining DNA and a plasmid (pFD116) without sequence homology to the *S. sanguinis* SK36 genome as the donor DNA. Natural transformation with this plasmid requires only its uptake, without genomic integration via homologous recombination. Similar to transformation using homologous DNA, the Δ*lytF* mutant (KP61) transformed on average 13-fold less efficiently than wild-type (KP52) when a plasmid was used as the transforming DNA (Fig. 3B). Consistent with the results presented above, ectopic complementation of *lytF* restored the transformation rate to that of the wild type. It thus seems likely that the absence of *lytF* affects the DNA uptake machinery in *S. sanguinis* since the effect on natural transformation persists even when homologous recombination is not involved.

### LytF influences the extracellular amount of the major competence pilin ComGC

**Fig 4:**
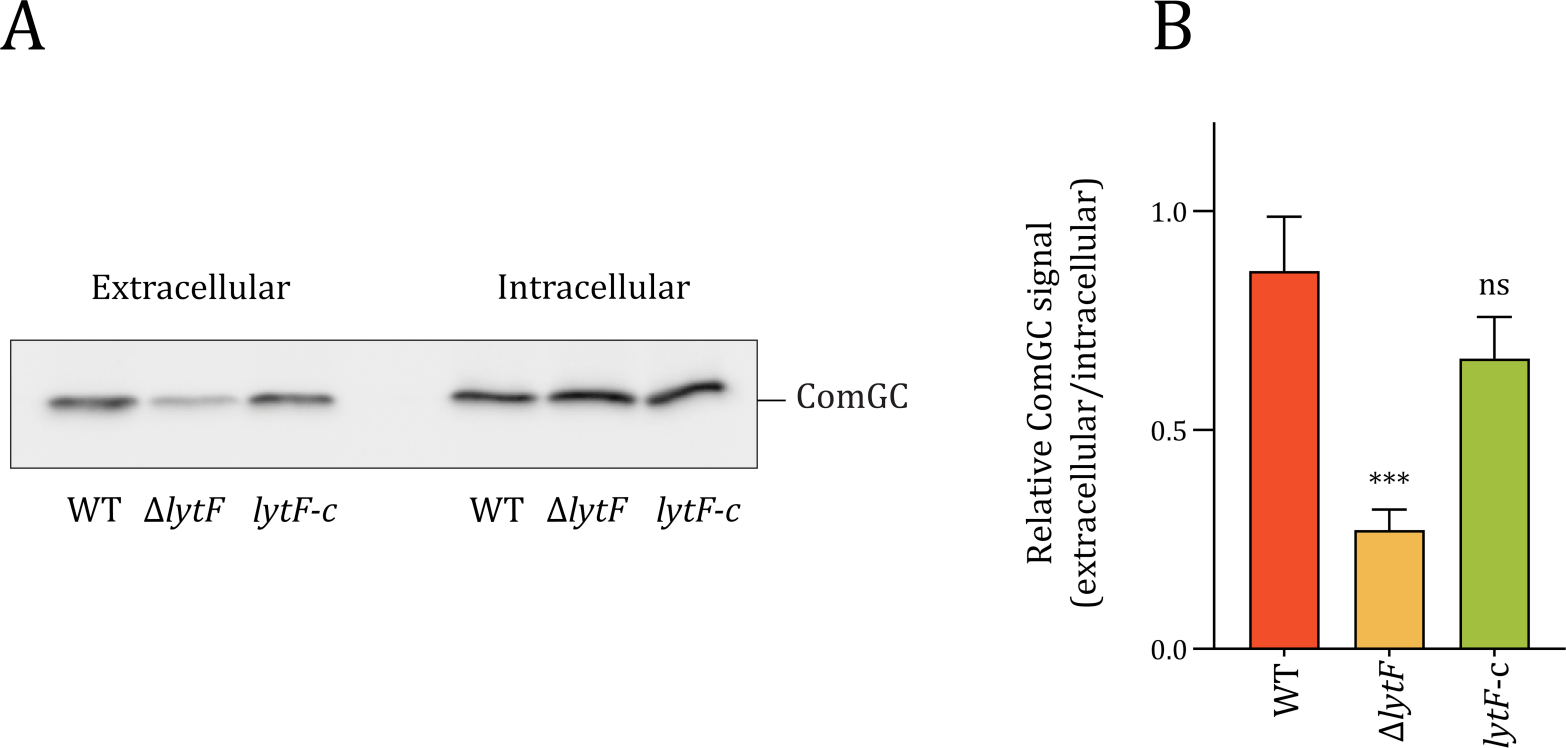
Effect of LytF on intracellular and extracellular levels of ComGC. Panel **A**: Immunoblots of ComGC-FLAG in intracellular and extracellular fractions of the wild type (RM32), a *lytF*-knockout (RM33), and a *lytF*-complementation strain (RM125). All the strains carry pFD116-P*comG-comGC-FLAG*. Pili were sheared from competent cells, and the cultures were split into extracellular and intracellular fractions by centrifugation. Anti-FLAG antibodies were applied to detect ComGC-FLAG. Panel **B**: Relative levels of ComGC-FLAG (extracellular/intracellular) measured from three different immunoblots using ImageJ (46). Statistical significance was determined using a one-way ANOVA and Dunnett’s test, ns = *P* > 0.05, *** = *P* ≤ 0.001. Results are based on data from three biologically independent experiments.

An important component of the DNA-uptake machinery in competent streptococci is the type IV competence pilus. In pneumococci, it has been shown to bind extracellular DNA and retract, bringing it closer to the cell (37, 45). ComGA and ComGB assemble at the membrane to produce the ComGC pilus filament that crosses the cell wall layer and extends into the extracellular space. To our knowledge, it is not known how the pilus is able to cross the peptidoglycan layer of the cell wall. Biørnstad, Ohnstad, and Håvarstein (31) speculated that CbpD of *S. thermophilus* contributes to an increase in transformation efficiency by making modifications to the cell wall that allow the pilus to penetrate the cell surface into the extracellular milieu. Following up on this idea, we compared the amount of competence pilus of LytF-proficient and -deficient cells. The strains RM32 (Δ*comC*) and RM33 (Δ*comC*, Δ*lytF*) carry a plasmid encoding a C-terminally FLAG-tagged ComGC, which allows for immunodetection of the major pilin. First, we established the dynamics of ComGC expression. Immunoblotting of whole-cell extracts of strain RM32 showed that ComGC-FLAG was expressed 10, 20, 30, and 40 minutes after competence induction, peaking at 30 minutes (Fig. S5). Therefore, samples for comparison of intra- and extracellular pilus levels were taken 30 minutes after competence induction. Notably, immunoblotting of cellular fractions demonstrated that the amount of ComGC-FLAG in the mutant strain (Δ*lytF*) was dramatically reduced extracellularly and slightly elevated intracellularly compared to the wild-type strain (Fig. 4A). For the complementation strain (*lytF*-c), both extracellular and intracellular fractions were similar to the wild type (Fig. 4A). The ratio between extracellular and intracellular levels of ComGC was found to be threefold higher in the wild-type strain than in the mutant and similar to the complementation strain (Fig. 4B). LytF did not affect production or stability of ComGC as the total amount was comparable across the strains (Fig. S6). These results suggest that the competence pilus relies on the presence of LytF to efficiently extend from the cell during assembly and could explain the Δ*lytF* mutant’s reduced ability for natural transformation.

**Fig 5:**
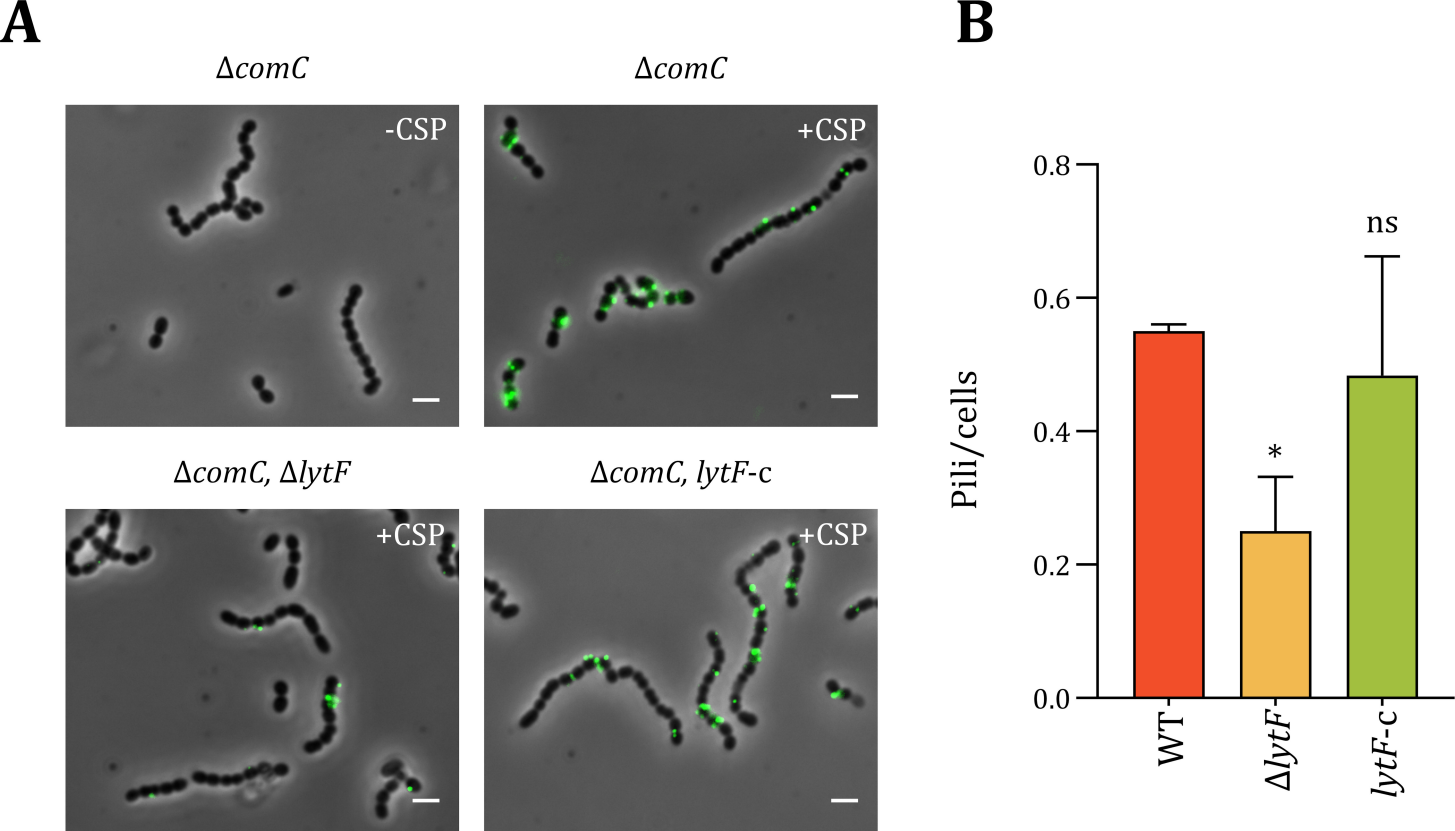
Detection of ComGC using immunofluorescence microscopy. Panel **A**: Competent cells of the strains RM32 (Δ*comC*), RM33 (Δ*comC*, Δ*lytF*), and RM125 (Δ*comC*, *lytF*-c) carrying the plasmid pFD116-*comGC*-FLAG were treated with anti-FLAG antibodies. A non-competence-induced sample of RM32 was included as a negative control. The images were processed using ImageJ, and the scale bars are 2 µm (46). Panel **B**: Counts of pili per cell as determined using MicrobeJ for three fluorescence microscopy images per strain, from two biologically independent experiments. The difference in means was determined to be statistically significant (*P* ≤ 0.05) between WT (RM32) and ΔlytF (RM33), but not (*P* > 0.05) between WT (RM32) *lytF*-c (RM125) using a one-way ANOVA and Dunnett test. Total number of cells counted: 5,587.

To confirm that the Δ*lytF* mutant indeed had fewer pili exposed on the cell surface, we performed immunofluorescence microscopy (targeting ComGC-FLAG) following the protocol used to visualize pili in *S. pneumoniae* (37). In the competence-induced Δ*comC* strain, ComGC-FLAG could be readily detected as fluorescent foci on the cell surface of numerous cells, whereas no foci were seen in the non-competent control cells (Fig. 5A). Transformation pili were also present on the cell surface of the competence-induced Δ*lytF* strain, but the number per cell was approximately half that observed in the wild type (Fig. 5B). Ectopic *lytF* expression rescued the wild-type phenotype. Together, these data corroborate the observation that loss of LytF reduces the number of extracellular pili.

## DISCUSSION

Competence-induced murein hydrolases in streptococci have previously been shown to be involved in a fratricide mechanism aimed at lysing sibling cells, or cells of closely related species, to release homologous DNA that can be taken up by the competent attacker cells and integrated into their genomes by homologous recombination (15, 16, 47). The prime example of this is the pneumococcal CbpD, which is expressed together with the cognate immunity protein ComM. Recently, it was shown that *Streptococcus dysgalactiae* in the pyogenic group also expresses an autolytic murein hydrolase (ScrM) and an immunity protein (ScrI) during competence, representing a system analogous to the pneumococcal CbpD-ComM (48). ScrM displayed high autolytic activity, but it remains to be shown experimentally if ScrM-ScrI is involved in fratricide. For species expressing LytF proteins, on the other hand, no LytF immunity protein has been identified (25), and it seems to have significantly less capacity to lyse target cells compared to CbpD and ScrM. Still, evidence that LytF is involved in fratricide comes from experiments showing that it increases gene transfer between *S. gordonii* strains and contributes to the release of eDNA in *S. gordonii* and *S. mutans* (25, 27, 28). Recently, it has also been shown to contribute to membrane vesicle release in *S. mutans* (49). In this work, we explored whether LytF functions as a fratricin in *S. sanguinis* SK36 by testing its ability to lyse non-competent cells as well as a mutant lacking a possible LytF immunity protein (SSA_RS01125, which shares some sequence similarity with the pneumococcal immunity protein ComM). Our observations did not support that *S. sanguinis* LytF functions to lyse sibling cells. Additionally, the lack of autolysis following competence induction of the Δ*SSA_RS01125* mutant (Fig. 1; Fig. S1) indicated that this mutant remained immune to self-produced LytF, suggesting that SSA_RS01125 is not an immunity factor. We should note that whether the expression of *lytF* is negatively affected upon *SSA_RS01125* deletion was not tested in the present study. Although SSA_RS01125 shares some sequence similarity to the pneumococcal ComM, *S. sanguinis* SK36 does not encode any ComM homologs (30). Besides, as LytF is unrelated to CbpD, it would be reasonable to assume that it requires an immunity mechanism other than pneumococci. However, when we treated non-competent cells with LytF, either as the purified enzyme (Fig. 2) or by an attacker strain (Fig. 3), neither resulted in significant cell lysis compared to competent cells. This suggests that *S. sanguinis* is intrinsically resistant to lysis by LytF and that protection against LytF might not be required. It should be noted, however, that we cannot rule out that LytF contributes to fratricide under *in vivo* conditions*,*which we were unable to reproduce in the current study.

A striking phenotype observed for some streptococci is that mutants lacking the gene encoding the competence-induced murein hydrolase are less transformable compared to their wild types when provided equal amounts of naked DNA, that is, there is no need for targeted cell lysis to make DNA available for transformation (29, 31, 32). While deletion of *cbpD* in *S. pneumoniae* R6 (Fig. S3) and *lytF* in *S. gordonii* NCTC 7868 did not significantly reduce the transformability compared to their wild-type counterparts (15, 25), a Δ*cbpD* mutant of *S. thermophilus* showed 17-fold reduced transformability (31). More severely, the rate of transformation in a *S. mutans* strain lacking LytF was less than 3% of wild-type levels (32). Cullin et al. reported that deletion of the *lytF* gene of *S. sanguinis* SK36 caused a fourfold reduction in transformability in this bacterium when inducing competence at the early exponential phase (29). In line with this, we found that the transformability of the wild-type *lytF*+ strain (KP52) was, on average, nine times higher than the transformability of the Δ*lytF* mutant (KP61) (Fig. 4A). Interestingly, the same effect was observed when using a plasmid as the transforming DNA (Fig. 4C), which suggests that the DNA-uptake system is somehow affected in the Δ*lytF* mutant. In addition, the transformation rate of a strain harboring a catalytically inactive version (LytF^C549A^) of the enzyme was comparable to that of the Δ*lytF* mutant (Fig. 4A). It suggests that LytF facilitates transformation by modifying the cell wall. Although we cannot exclude the possibility that the C549A substitution induces significant changes to the protein structure, a similar substitution in other CHAP domains has been shown not to influence the overall stability or solubility of this domain (50, 51). To our surprise, neither addition of purified His_6_-LytF nor LytF derived from co-culturing with a competent *lytF*+-strain (KP52) restored the transformability of a *lytF*^-^-strain (KP54) (Fig. 3A). This shows that LytF is unable to complement a Δ*lytF* mutant when added from the outside. Ectopic expression of *lytF*, on the other hand, restored transformability to wild-type levels (Fig. 3A). As LytF contains a signal sequence for secretion, these results could mean that LytF is secreted to and is performing its role in the periplasmic space. This could explain the lack of observed lytic activity by extracellular addition.

Streptococcal DNA uptake relies on the type IV competence pili, which act as fishing lines for extracellular DNA (37, 45). During competence, newly synthesized type IV pili alternately extend and retract, presumably functioning as a grappling hook that attaches to extracellular DNA and pulls it across the capsule and peptidoglycan layer. The diameter of the pores of the inner peptidoglycan layer of gram-positive bacteria has been estimated to be around 6 nm (38). This is also the estimated diameter of the type IV competence pilus (37). Recently, the DNA-binding site of the *S. sanguinis* com-pilus was identified within the ComGDEFG tip complex (35). In this model, dsDNA binds at the interface between ComGD and ComGF, extending beyond its surface, effectively increasing the overall diameter of the complex to at least 8 nm. Consequently, widening of existing pores or creation of pilus-specific pores would be required to make room for the pilus-DNA structure. During assembly of other trans-envelope structures (e.g., bacterial flagella or secretion systems) that are too large to pass through the natural pores of the peptidoglycan sacculus, bacteria are known to produce dedicated peptidoglycan hydrolases, which are critical to create the needed space in peptidoglycan (41, 52). For example, in *Neisseria gonorrhoeae,* AtlA degrades peptidoglycan, enabling the assembly of a DNA-releasing type IV secretion system without inducing cell lysis (53). Our results established that the *S. sanguinis* Δ*lytF* mutant is less piliated than the wild type, and ectopic expression of *lytF* from its native promoter restored piliation to wild-type levels (Fig. 4 and 5). We therefore suggest that the Δ*lytF* mutant shows a reduced ability to transform when naked DNA is available because it has fewer extracellular type IV pili to “catch” the DNA. We also showed that normal transformation rates most probably depended specifically on the muralytic activity of LytF (Fig. 3A), suggesting that it aids the competence pilus in entering the extracellular space by making specific cuts in the peptidoglycan layer. The peptidoglycan hydrolase may somehow associate with the pilus and degrade peptidoglycan before or as it extends across the cell wall. Alternatively, LytF may degrade peptidoglycan to enlarge random pre-existing pores, thereby increasing the likelihood of successful pili penetration. Whereas the competence pilus is essential for transformation in *S. sanguinis* (34), LytF is not, indicating that pilus extrusion is not entirely dependent on LytF. Our immunoblot (Fig. 4) and microscopy images (Fig. 5) showed that, although to a lesser extent than in the wild type, ComGC is detected extracellularly on competent Δ*lytF* cells, suggesting that the pilus is severely hindered but not completely blocked from exiting the cell.

Several streptococcal species encode a LytF-like protein, with the only structural difference between them being the number of Bsp domains. In *S. mutans*, LytF is an autolytic enzyme that specifically triggers lysis in CSP-responsive cells and is important for eDNA release during biofilm formation (26, 28). It has been proposed that this function of LytF is analogous to eukaryotic apoptosis, serving as a response to environmental stress for the overall benefit of the population (26). But lack of LytF has also been shown to reduce transformability in this species dramatically (Eaton Jacques 2010). In *S. gordonii*, which is more closely related to *S. sanguinis*, LytF strongly increases the rate of gene transfer between two different *S. gordonii* strains during co-cultivation without causing autolysis, but minimally affects the transformation rate in pure cultures (25, 27). Our results for LytF from *S. sanguinis* SK36 do not entirely align with those of *S. mutans* LytF or *S. gordonii* LytF, as the former is autolytic while the latter does not affect transformability. Although structurally similar, the LytF hydrolases from different species seem to perform unique roles in the transformation process.

As outlined above, there are data showing that competence-induced peptidoglycan hydrolases in streptococci function as fratricins, facilitators of DNA uptake, or both. How can these findings be reconciled? Since solid experimental data support both functions, there seem to be two possible explanations. Either these peptidoglycan hydrolases play different roles in different streptococcal species or they have a dual function. As no evidence of fratricide was found for *S. sanguinis* LytF, it seems that, at least for this enzyme, the prior hypothesis is more likely. There does not seem to be a clear pattern for predicting which of the two functions the different competence-induced hydrolases perform. Both LytF (*S. sanguinis*, *S. mutans*) and CbpD-like (*S. thermophilus*, *S. suis*) enzymes have been reported to affect the transformability, but not all of either variant (15, 25, 29, 31–33). The function is also independent of phylogenetic group classification, as the murein hydrolases of *S. pneumoniae* (CbpD), *S. gordonii* (LytF), and *S. sanguinis* (LytF), all belonging to the mitis group, differ in function (15, 25).

In this study, we could not find evidence supporting LytF to function as a fratricin in *S. sanguinis* SK36, but instead found that the expression of LytF influences the transformability of *S. sanguinis* by aiding the competence pilus in entering the extracellular environment.

## MATERIALS AND METHODS

### Bacterial strains and growth conditions

Bacterial strains and species used in this study are listed in Table S1. Todd-Hewitt (TH) broth was used to grow *S. sanguinis* SK36 and its derivatives at 37°C without shaking, except when stated otherwise. When cultivated on TH agar plates, they were grown in anaerobic conditions (<1% O_2_), obtained using AnaeroGen sachets from Oxoid in a sealed container. The *E. coli* strains were grown aerobically in lysogeny broth (LB) or on lysogeny agar (LA) plates at 37°C. When required, TH media were supplemented with 400 µg/mL kanamycin or 200 µg/mL streptomycin. For plasmid retention, 100 µg/mL and 60 µg/mL spectinomycin were used for *S. sanguinis* and *E. coli*, respectively.

### Competence induction and natural transformation

Competence was induced at OD_550_ = 0.2–0.4 using 250 ng/mL CSP (H_2_N-DLRGVPNPWGWIFGR-COOH). The transforming DNA was added to a final concentration of 250 ng/mL, and the transforming culture was incubated for 2 hours at 37°C before 50–100 µL were plated on TH agar containing the selective antibiotic.

### Construction of mutant strains

Primers used to construct the mutants used in this study are listed in Table S2. The Janus cassette was used to create streptococcal mutants (54). The cassette allows for both the selection of its acquisition and its loss. It comprises a kanamycin resistance gene (KmR) and a counter-selectable marker encoding a wild-type streptomycin-sensitive *rpsL* gene (*rpsL*+) that confers dominant streptomycin sensitivity in a streptomycin-resistant background. Hence, to use the Janus cassette for mutant construction in *S. sanguinis* SK36, we made a streptomycin-resistant mutant of the SK36 strain that contains mutations in its native *rpsL* allele conferring streptomycin resistance. This was achieved by selection on TH agar plates containing 1 mg/mL of streptomycin, giving rise to the streptomycin-resistant mutant KP36.

To delete the *comC* gene of the KP36 strain, the Janus cassette was amplified from genomic DNA obtained from *S. pneumoniae* strain SPH131 using the kp211/kp212 primers. Regions flanking *comC* (approximately 1,000 bp) were amplified from genomic DNA of strain SK36 using primers kp207/kp208 and kp209/kp210. The three PCR products were fused by overlap extension PCR using primers kp207/kp210 (55). The resulting amplicon was introduced into KP36 through natural transformation. The Janus cassette was subsequently removed by transformation with a PCR construct consisting of only the fused flanking regions, followed by selection on streptomycin-containing TH-agar plates. This resulted in the mutant KP52, in which only the coding region of the *comC* gene had been excised. All genetic modifications introduced to *S. sanguinis* were performed using this approach. Strains RM88, RM104, and RM133 were created by removing the Janus cassette, replacing *lytF* with PCR constructs comprised of the *lytF*-flanking regions fused to the modified variants of the gene (*lytF-sfGFP*, *his-lytF*, and *lytF*_C549A_, respectively). Similarly, RM139 and RM140 were constructed by replacing a Janus cassette inserted downstream of *comX* with a *comX-sfGFP* construct. Transformants were verified by colony PCR and Sanger sequencing (GATC, Eurofins).

For β-galactosidase assays, *lacZ* was introduced to *S. sanguinis* by placing it in the *hirL* locus (*SSA_RS09615*), similarly to the approach used for *S. pneumoniae* (42). A Δ*hirL*::janus cassette was constructed by amplifying janus using primers Kan484F/RpsL41R and gDNA from RH426 as template. The *hirL* upstream and downstream regions were amplified from genomic DNA of *S. sanguinis* SK36 using primer pairs ds703/ds704 and ds705/ds706, respectively. Then the three amplicons were fused by using primer pair ds703/ds706. A Δjanus::P3-*lacZ* amplicon was used to replace janus in the *hirL* locus with P3-*lacZ* (transcription of *lacZ* is driven by a constitutive synthetic P3 promoter with sequence 5′-TTGCACTGTCCCCCTGGTATAATAACTATACATGCAAGATCTAAAT-3′). P3-*lacZ* was amplified from genomic DNA derived from strain RH2 using primers ds711 and ds712. For the replacement of Janus, the P3-*lacZ* amplicon was fused to the *hirL* upstream and downstream regions. The upstream region was amplified using primer pair ds703/ds713, and the downstream region using primers ds706/ds714 and *S. sanguinis* SK36 gDNA as template. Finally, primers ds703 and ds706 were used to fuse the three amplicons and produce Δjanus::P3-*lacZ*.

The pFD116-P*comGA-comGC*-FLAG plasmid was constructed by first amplifying the promoter region (156 bp upstream of *comGA*) of the *comG* operon and the *comGC* gene from *S. sanguinis* SK36. Primer pairs rm032/rm035 and rm033/rm034 were used, respectively. Primer rm034 contains a FLAG tag-encoding sequence. The amplified fragments were joined by overlap extension PCR, cleaved with SpeI and NcoI, and ligated into pFD116. Next, the ligation reaction was transformed into chemically competent *E. coli* cells by heat shock at 42°C. Finally, the plasmid sequence was verified by Sanger sequencing (GATC, Eurofins) and transformed into *S. sanguinis* by natural transformation, as described above.

### Purification of His_6_-LytF

His_6_-LytF was purified from the supernatant of a competent *S. sanguinis* culture (RM104), carrying the *lytF* gene fused with 6x-*his*, using immobilized metal affinity chromatography (IMAC). A 500 mL culture of OD_550_ = 0.2 was induced to competence and incubated at 37°C for 1 hour. After centrifugation at 5,000 × g for 10 minutes, the supernatant containing His_6_-LytF was filtered using a 0.45 µm filter. The filtrate was then passed through a 1 mL HisTrap HP column (Cytiva Life Sciences) with the Äktaprime plus system (GE Healthcare). The column was washed with 10 column volumes of 10 mM Tris-HCl (pH 7.4), 150 mM NaCl, 20 mM imidazole before His_6_-LytF was eluted using a linear gradient of imidazole from 20 to 500 mM in the same buffer conditions. The eluate was dialyzed using a buffer of 10 mM Tris-HCl, 150 mM NaCl, pH = 7.4, for 1 hour at room temperature. The presence and purity of the protein were assessed by SDS-PAGE electrophoresis (Fig. S1) before it was stored at −80°C.

### Zymography of His_6_-LytF activity

Zymogram analyses were performed based on the protocol described by Leclerc and Asselin (1989). Briefly, 75 mL of the substrate culture (KP52) was grown to OD_550_ = 0.8. The cells were collected by centrifugation at 5,000 × *g*, resuspended in 1.25 mL of 1.5 M Tris-HCl, pH 8.8, and heat-treated for 10 minutes at 95°C before being included in gel casting. SDS-PAGE was performed as described by Laemmli (1970) using a 4% stacking gel and a 10% separation gel mixed with the suspension of the substrate culture. The cell lysate samples were prepared from 10 mL cultures of KP52, KP61, and RM104. After competence induction, the cultures were incubated at 37°C for 30 minutes, harvested by centrifugation at 5,000 × *g*, resuspended in 200 µL PBS, and lysed using the FastPrep−24 homogenizer (MP Bio). The cell lysate samples were normalized based on total protein concentration. A total of 95 µg and 0.5 µg of the cell lysate samples and purified His_6_-LytF were loaded onto the gel, respectively. Following electrophoresis, the gel was washed twice in deionized water for 30 minutes before the refolding buffer was added (100 mM NaCl, 1 mM MgCl_2_, 0.5% Triton X‐100, 20 mM Tris‐HCl, pH 7.4). The gel was incubated in the refolding buffer until lytic zones appeared.

### β-galactosidase assay

Exponentially growing *S. sanguinis* SK36 mutants expressing *lacZ* constitutively were diluted to OD_550_ = 0.05 in a final volume of 5 mL TH broth. The bacteria were grown to OD_550_ = 0.3 and induced to competence. Uninduced cultures were grown in parallel as a negative control. For co-cultivation experiments, 2.5 mL of the attacker and 2.5 mL of the target cells were mixed at OD_550_ = 0.3, and CSP was added to induce competence in the attacker cells. After incubation at 37°C for 60 minutes, the β-galactosidase activity released to the growth medium and total β-galactosidase activity in the culture (growth medium and intracellular) were quantified. Three milliliters from each cell culture was used as follows: 1 mL was sterile filtered through a 0.2 µm filter to obtain cell-free culture supernatants, 1 mL of the cell culture was lysed using bead beating with 0.5 *g* ≤ 106 µm glass beads (Sigma) for 3 × 20 seconds at 6.5 m/s, and 1 mL was used to measure OD_550_. The β-galactosidase activity was detected by adding 960 µL culture supernatant or 200 µL clarified cell lysate to 240 µL 5× Z-buffer (5 mM MgCl_2_, 250 mM β-mercaptoethanol, 50 mM KCl, 0.3 M Na_2_HPO_4_, 0.2 M NaH_2_PO_4_) containing 4 mg/mL o-nitrophenyl-β-D-galactopyranoside (ONPG). For the cell lysate samples, 760 µL TH medium was added to a final volume of 1,200 µL. The samples were incubated at 37°C for 90 minutes before the reaction was stopped by adding 500 µL of 1 M Na_2_CO_3_. ONPG is hydrolyzed by β-galactosidase to galactose and ortho-nitrophenol; the absorption of the latter was measured at 420 nm. The β-galactosidase activity was calculated according to Miller et al. (56).

### SYTOX Green nucleic acid stain assay

The Δ*comC* mutant (KP52) was grown to OD_550_ ~ 0.2 in C-medium (57) before being diluted to OD_550_ = 0.05. Here, C-medium was used instead of TH as its lack of color ensures minimal interference with the fluorescent signal. The culture was transferred to a transparent bottom black polystyrene 96-well plate (Merck), and SYTOX Green Nucleic Acid stain (ThermoFisher) was added to a final concentration of 2 µM. The stain fluoresces when binding nucleic acids but is unable to cross intact cell membranes. Optical density (550 nm) and fluorescence (excitation at 485 nm and emission at 535 nm) were measured using the HIDEX Sense microplate reader. At OD_550_ = 0.2, a final concentration of 5 µg/mL His-LytF was added to the culture.

### Transformation efficiency assays

The transformation efficiency assays were performed by natural transformation using a Δ*SSA_RS01125::*Janus cassette. Following transformation, the cultures were serially diluted and plated on TH plates with and without kanamycin. The plates were incubated anaerobically overnight, and colonies were counted the next day. Colonies on the antibiotic- and non-antibiotic plates were used to estimate transformant and total cell count, respectively. The transformation efficiency was calculated as the number of transformants divided by the total cell count and was based on at least three repetitions of each experiment. Only plates containing between 30 and 300 colonies were included in the calculations. To assess at what OD_550_ *S. sanguinis* SK36 transformed the most efficiently, the assay was performed as described above, except samples from a growing culture were taken for every timepoint, representing various OD_550_ values ranging from 0.05 to 0.9 with increments of 0.1. For the co-cultivation assay, a culture consisting of KP52 and KP54 in equal amounts was prepared in parallel right before competence induction and transformed using a Δ*SSA_RS01125::aad9* cassette.

### Western blotting

Competence was induced in a 5 mL culture at OD_550_ = 0.2, which was then incubated for 30 minutes at 37°C. The pilus appendages were mechanically detached from cells by vortexing for the last 10 minutes of incubation. Cells and supernatant were split by centrifugation at 4,000 × *g* for 10 minutes. After decanting the supernatant containing the sheared pili, the cells were washed once with PBS and lysed in 200 µL PBS using the FastPrep−24 homogenizer (MP Bio). All samples were normalized based on total protein amounts of the whole-cell extracts dissolved in 1% SDS estimated by measuring the A280 nm. Following SDS-PAGE (15% resolving gel) and transfer of the separated proteins onto a methanol-activated PVDF membrane using electroblotting, the membrane was blocked in 5% skim milk in TBS-T (wt/vol) at room temperature for 1 hour and then at 4°C O/N. The membrane was incubated with anti-FLAG antibodies (1:4,000, ThermoFisher) and anti-rabbit antibodies (1:4,000, HRP, ThermoFisher) for 1 hour each and was washed with TBS-T (3 × 10 min) after each incubation. Finally, secondary antibodies associated with the membrane were revealed using the SuperSignal West Pico PLUS Chemiluminescent Substrate (ThermoFisher) and the Azure c400 Imaging System (Azure Biosystems).

For the time-series experiment, cultures were incubated for 10–40 minutes with increments of 10 following competence induction. A non-induced culture was included as a negative control (time 0). SDS-PAGE and blotting were performed as described above.

### Microscopy

Fluorescence microscopy for visual detection of ComGC was performed as outlined by Laurenceau et al. (37) with minor modifications. Briefly, the cells were induced to competence and incubated for 30 minutes. They were then harvested and resuspended in 500 µL PBS. A 50 µL aliquot of this suspension was allowed to adhere to a poly-L-lysine slide for 5 minutes. This and all the following steps were conducted in a humidity chamber. The cells were fixed with 3.7% formaldehyde in PBS, blocked with 1% BSA for 5 minutes, then incubated with anti-FLAG antibodies (1:4,000, ThermoFisher) for 1 hour, followed by an incubation with anti-rabbit antibodies (1:4,000, Alexa Fluor 488, ThermoFisher) for another hour. In between these steps, the cells were washed with PBS. Images were taken with a Zeiss LSM700 microscope and analyzed using ImageJ (46).

For live/dead imaging, the BacLight Live/Dead staining kit (Invitrogen) was used to stain planktonic cultures as described by the manufacturer. Images were taken using a 555 nm laser line for the excitation of propidium iodide, and a 488 nm laser line was applied for the excitation of SYTO 9. Zen software (ZEN Zeiss Lite) and the ImageJ plugin MicrobeJ were used for image analysis (46, 58).

To determine the fraction of competent cells after induction, cultures of RM139 (Δ*comC*, *comX-sfGFP*) and RM140 (Δ*comC*, Δ*lytF*, *comX-sfGFP*) were induced to competence as described previously and incubated at 37°C for 30 minutes. A non-induced culture of RM139 was used as a negative control. The cells were then harvested by centrifugation at 5,000 × *g* for 5 minutes, before being washed and resuspended in PBS. Images were taken with a Zeiss LSM700 microscope, and the ImageJ plugin MicrobeJ was used for image analysis (46, 58).

## SUPPLEMENTAL MATERIAL

Supplemental Material jb.00118-26-s0001.docx Tables S1 to S3; Fig. S1 to S7.

ASM does not own the copyrights to Supplemental Material that may be linked to, or accessed through, an article. The authors have granted ASM a non-exclusive, world-wide license to publish the Supplemental Material files. Please contact the corresponding author directly for reuse.

## References

[1] Johnston C, Martin B, Fichant G, Polard P, Claverys J-P. 2014. Bacterial transformation: distribution, shared mechanisms and divergent control. Nat Rev Microbiol 12:181–196. doi:10.1038/nrmicro3199 24509783

[2] Håvarstein LS, Coomaraswamy G, Morrison DA. 1995. An unmodified heptadecapeptide pheromone induces competence for genetic transformation in Streptococcus pneumoniae. Proc Natl Acad Sci USA 92:11140–11144. doi:10.1073/pnas.92.24.11140 7479953 PMC40587

[3] Fontaine L, Boutry C, de Frahan MH, Delplace B, Fremaux C, Horvath P, Boyaval P, Hols P. 2010. A novel pheromone quorum-sensing system controls the development of natural competence in Streptococcus thermophilus and Streptococcus salivarius. J Bacteriol 192:1444–1454. doi:10.1128/JB.01251-09 20023010 PMC2820839

[4] Pestova EV, Håvarstein LS, Morrison DA. 1996. Regulation of competence for genetic transformation in Streptococcus pneumoniae by an auto-induced peptide pheromone and a two-component regulatory system. Mol Microbiol 21:853–862. doi:10.1046/j.1365-2958.1996.501417.x 8878046

[5] Håvarstein LS, Gaustad P, Nes IF, Morrison DA. 1996. Identification of the streptococcal competence-pheromone receptor. Mol Microbiol 21:863–869. doi:10.1046/j.1365-2958.1996.521416.x 8878047

[6] Fontaine L, Wahl A, Fléchard M, Mignolet J, Hols P. 2015. Regulation of competence for natural transformation in streptococci. Infect Genet Evol 33:343–360. doi:10.1016/j.meegid.2014.09.010 25236918

[7] Shanker E, Morrison DA, Talagas A, Nessler S, Federle MJ, Prehna G. 2016. Pheromone recognition and selectivity by ComR proteins among Streptococcus species. PLoS Pathog 12:e1005979. doi:10.1371/journal.ppat.1005979 27907154 PMC5131902

[8] Kilian M, Poulsen K, Blomqvist T, Håvarstein LS, Bek-Thomsen M, Tettelin H, Sørensen UBS. 2008. Evolution of Streptococcus pneumoniae and its close commensal relatives. PLoS One 3:e2683. doi:10.1371/journal.pone.0002683 18628950 PMC2444020

[9] Blomqvist T, Kilian M, Håvarstein LS. 2007. Biological active peptides in streptococci, p 25–59. In Hackenbeck R, Chhatwal S (ed), Molecular biology of streptococci. Taylor & Francis.

[10] Peterson S, Cline RT, Tettelin H, Sharov V, Morrison DA. 2000. Gene expression analysis of the Streptococcus pneumoniae competence regulons by use of DNA microarrays. J Bacteriol 182:6192–6202. doi:10.1128/JB.182.21.6192-6202.2000 11029442 PMC94756

[11] Lee MS, Morrison DA. 1999. Identification of a new regulator in Streptococcus pneumoniae linking quorum sensing to competence for genetic transformation. J Bacteriol 181:5004–5016. doi:10.1128/JB.181.16.5004-5016.1999 10438773 PMC93990

[12] Berg KH, Biørnstad TJ, Johnsborg O, Håvarstein LS. 2012. Properties and biological role of streptococcal fratricins. Appl Environ Microbiol 78:3515–3522. doi:10.1128/AEM.00098-12 22407687 PMC3346361

[13] Kausmally L, Johnsborg O, Lunde M, Knutsen E, Håvarstein LS. 2005. Choline-binding protein D (CbpD) in Streptococcus pneumoniae is essential for competence-induced cell lysis. J Bacteriol 187:4338–4345. doi:10.1128/JB.187.13.4338-4345.2005 15968042 PMC1151764

[14] Guiral S, Mitchell TJ, Martin B, Claverys JP. 2005. Competence-programmed predation of noncompetent cells in the human pathogen Streptococcus pneumoniae: genetic requirements. Proc Natl Acad Sci USA 102:8710–8715. doi:10.1073/pnas.0500879102 15928084 PMC1150823

[15] Johnsborg O, Eldholm V, Bjørnstad ML, Håvarstein LS. 2008. A predatory mechanism dramatically increases the efficiency of lateral gene transfer in Streptococcus pneumoniae and related commensal species. Mol Microbiol 69:245–253. doi:10.1111/j.1365-2958.2008.06288.x 18485065

[16] Håvarstein LS, Martin B, Johnsborg O, Granadel C, Claverys JP. 2006. New insights into the pneumococcal fratricide: relationship to clumping and identification of a novel immunity factor. Mol Microbiol 59:1297–1307. doi:10.1111/j.1365-2958.2005.05021.x 16430701

[17] Minhas V, Domenech A, Synefiaridou D, Straume D, Brendel M, Cebrero G, Liu X, Costa C, Baldry M, Sirard JC, Perez C, Gisch N, Hammerschmidt S, Håvarstein LS, Veening JW. 2023. Competence remodels the pneumococcal cell wall exposing key surface virulence factors that mediate increased host adherence. PLoS Biol 21:e3001990. doi:10.1371/journal.pbio.3001990 36716340 PMC9910801

[18] Straume D, Stamsås GA, Salehian Z, Håvarstein LS. 2017. Overexpression of the fratricide immunity protein ComM leads to growth inhibition and morphological abnormalities in Streptococcus pneumoniae. Microbiology (Reading) 163:9–21. doi:10.1099/mic.0.000402 27902435

[19] Bergé MJ, Mercy C, Mortier-Barrière I, VanNieuwenhze MS, Brun YV, Grangeasse C, Polard P, Campo N. 2017. A programmed cell division delay preserves genome integrity during natural genetic transformation in Streptococcus pneumoniae. Nat Commun 8:1621. doi:10.1038/s41467-017-01716-9 29158515 PMC5696345

[20] Juillot D, Billaudeau C, Mortier-Barrière I, Barbotin A, Lablaine A, Polard P, Campo N, Carballido-López R. 2025. Transient inhibition of cell division in competent pneumococcal cells results from deceleration of the septal peptidoglycan complex. Nat Commun 16:5666. doi:10.1038/s41467-025-60600-z 40595580 PMC12214887

[21] Layec S, Decaris B, Leblond-Bourget N. 2008. Characterization of proteins belonging to the CHAP-related superfamily within the Firmicutes. J Mol Microbiol Biotechnol 14:31–40. doi:10.1159/000106080 17957108

[22] Pérez-Dorado I, Galan-Bartual S, Hermoso JA. 2012. Pneumococcal surface proteins: when the whole is greater than the sum of its parts. Mol Oral Microbiol 27:221–245. doi:10.1111/j.2041-1014.2012.00655.x 22759309

[23] Eldholm V, Johnsborg O, Straume D, Ohnstad HS, Berg KH, Hermoso JA, Håvarstein LS. 2010. Pneumococcal CbpD is a murein hydrolase that requires a dual cell envelope binding specificity to kill target cells during fratricide. Mol Microbiol 76:905–917. doi:10.1111/j.1365-2958.2010.07143.x 20384696

[24] Straume D, Piechowiak KW, Olsen S, Stamsås GA, Berg KH, Kjos M, Heggenhougen MV, Alcorlo M, Hermoso JA, Håvarstein LS. 2020. Class A PBPs have a distinct and unique role in the construction of the pneumococcal cell wall. Proc Natl Acad Sci USA 117:6129–6138. doi:10.1073/pnas.1917820117 32123104 PMC7084106

[25] Berg KH, Ohnstad HS, Håvarstein LS. 2012. LytF, a novel competence-regulated murein hydrolase in the genus Streptococcus. J Bacteriol 194:627–635. doi:10.1128/JB.06273-11 22123253 PMC3264080

[26] Dufour D, Lévesque CM. 2013. Cell death of Streptococcus mutans induced by a quorum-sensing peptide occurs via a conserved streptococcal autolysin. J Bacteriol 195:105–114. doi:10.1128/JB.00926-12 23104806 PMC3536168

[27] Xu Y, Kreth J. 2013. Role of LytF and AtlS in eDNA Release by Streptococcus gordonii. PLoS One 8:e62339. doi:10.1371/journal.pone.0062339 23638042 PMC3634736

[28] Nagasawa R, Yamamoto T, Utada AS, Nomura N, Obana N. 2020. Competence-stimulating-peptide-dependent localized cell death and extracellular DNA production in Streptococcus mutans biofilms. Appl Environ Microbiol 86:e02080-20. doi:10.1128/AEM.02080-20 32948520 PMC7657630

[29] Cullin N, Redanz S, Lampi KJ, Merritt J, Kreth J. 2017. Murein hydrolase LytF of Streptococcus sanguinis and the ecological consequences of competence development. Appl Environ Microbiol 83:e01709-17. doi:10.1128/AEM.01709-17 28986373 PMC5717204

[30] Rodriguez AM, Callahan JE, Fawcett P, Ge X, Xu P, Kitten T. 2011. Physiological and molecular characterization of genetic competence in Streptococcus sanguinis. Mol Oral Microbiol 26:99–116. doi:10.1111/j.2041-1014.2011.00606.x 21375701 PMC3076142

[31] Biørnstad TJ, Ohnstad HS, Håvarstein LS. 2012. Deletion of the murein hydrolase CbpD reduces transformation efficiency in Streptococcus thermophilus. Microbiology (Reading, Engl) 158:877–885. doi:10.1099/mic.0.056150-0 22241050

[32] Eaton RE, Jacques NA. 2010. Deletion of competence-induced genes over-expressed in biofilms caused transformation deficiencies in Streptococcus mutans. Mol Oral Microbiol 25:406–417. doi:10.1111/j.2041-1014.2010.00589.x 21040514

[33] Zhu Y, Ma J, Zhang Y, Zhong X, Bai Q, Dong W, Pan Z, Liu G, Zhang C, Yao H. 2021. CrfP, a fratricide protein, contributes to natural transformation in Streptococcus suis. Vet Res 52:50. doi:10.1186/s13567-021-00917-x 33762005 PMC7992943

[34] Mom J, Chouikha I, Valette O, Pieulle L, Pelicic V. 2024. Systematic functional analysis of the Com pilus in Streptococcus sanguinis: a minimalistic type 4 filament dedicated to DNA uptake in monoderm bacteria. mBio 15:e0266723. doi:10.1128/mbio.02667-23 38095871 PMC10790768

[35] Mom J, Valette O, Pieulle L, Pelicic V. 2025. Unraveling the molecular mechanisms of DNA capture by the Com pilus in naturally transformable monoderm bacteria. mBio 16:e0085125. doi:10.1128/mbio.00851-25 40407325 PMC12153270

[36] Pelicic V. 2023. Mechanism of assembly of type 4 filaments: everything you always wanted to know (but were afraid to ask). Microbiology (Reading) 169:001311. doi:10.1099/mic.0.001311 36947586 PMC10191384

[37] Laurenceau R, Péhau-Arnaudet G, Baconnais S, Gault J, Malosse C, Dujeancourt A, Campo N, Chamot-Rooke J, Le Cam E, Claverys J-P, Fronzes R. 2013. A type IV pilus mediates DNA binding during natural transformation in Streptococcus pneumoniae. PLoS Pathog 9:e1003473. doi:10.1371/journal.ppat.1003473 23825953 PMC3694846

[38] Pasquina-Lemonche L, Burns J, Turner RD, Kumar S, Tank R, Mullin N, Wilson JS, Chakrabarti B, Bullough PA, Foster SJ, Hobbs JK. 2020. The architecture of the Gram-positive bacterial cell wall. Nature 582:294–297. doi:10.1038/s41586-020-2236-6 32523118 PMC7308169

[39] Muschiol S, Balaban M, Normark S, Henriques-Normark B. 2015. Uptake of extracellular DNA: competence induced pili in natural transformation of Streptococcus pneumoniae. Bioessays 37:426–435. doi:10.1002/bies.201400125 25640084 PMC4405041

[40] Koraimann G. 2003. Lytic transglycosylases in macromolecular transport systems of Gram-negative bacteria. Cell Mol Life Sci 60:2371–2388. doi:10.1007/s00018-003-3056-1 14625683 PMC11138577

[41] Scheurwater EM, Burrows LL. 2011. Maintaining network security: how macromolecular structures cross the peptidoglycan layer. FEMS Microbiol Lett 318:1–9. doi:10.1111/j.1574-6968.2011.02228.x 21276045

[42] Steinmoen H, Knutsen E, Håvarstein LS. 2002. Induction of natural competence in Streptococcus pneumoniae triggers lysis and DNA release from a subfraction of the cell population. Proc Natl Acad Sci USA 99:7681–7686. doi:10.1073/pnas.112464599 12032343 PMC124321

[43] Shields RC, Burne RA. 2016. Growth of Streptococcus mutans in biofilms alters peptide signaling at the sub-population level. Front Microbiol 7:1075. doi:10.3389/fmicb.2016.01075 27471495 PMC4946182

[44] Slager J, Kjos M, Attaiech L, Veening JW. 2014. Antibiotic-induced replication stress triggers bacterial competence by increasing gene dosage near the origin. Cell 157:395–406. doi:10.1016/j.cell.2014.01.068 24725406

[45] Lam T, Ellison CK, Eddington DT, Brun YV, Dalia AB, Morrison DA. 2021. Competence pili in Streptococcus pneumoniae are highly dynamic structures that retract to promote DNA uptake. Mol Microbiol 116:381–396. doi:10.1111/mmi.14718 33754381

[46] Rueden CT, Schindelin J, Hiner MC, DeZonia BE, Walter AE, Arena ET, Eliceiri KW. 2017. ImageJ2: ImageJ for the next generation of scientific image data. BMC Bioinformatics 18:529. doi:10.1186/s12859-017-1934-z 29187165 PMC5708080

[47] Wei H, Håvarstein LS. 2012. Fratricide is essential for efficient gene transfer between pneumococci in biofilms. Appl Environ Microbiol 78:5897–5905. doi:10.1128/AEM.01343-12 22706053 PMC3406168

[48] Mårli MT, Arntzen MØ, Allred JA, Schultheiss AT, Oppegaard O, Kjos M, Straume D. 2025. Self-immunity towards a novel competence-induced streptococcal peptidoglycan hydrolase is mediated by a fem-transferase-like protein. Mol Microbiol 123:516–530. doi:10.1111/mmi.15361 40156435

[49] Nagasawa R, Nomura N, Obana N. 2023. Identification of a novel gene involved in cell-to-cell communication-induced cell death and eDNA production in Streptococcus mutans. Microbes Environ 38:ME22085. doi:10.1264/jsme2.ME22085 37302844 PMC10308232

[50] Sanz-Gaitero M, Keary R, Garcia-Doval C, Coffey A, van Raaij MJ. 2014. Crystal structure of the lytic CHAP(K) domain of the endolysin LysK from Staphylococcus aureus bacteriophage K. Virol J 11:133. doi:10.1186/1743-422X-11-133 25064136 PMC4126393

[51] Bartual SG, Straume D, Stamsås GA, Muñoz IG, Alfonso C, Martínez-Ripoll M, Håvarstein LS, Hermoso JA. 2014. Structural basis of PcsB-mediated cell separation in Streptococcus pneumoniae. Nat Commun 5:3842. doi:10.1038/ncomms4842 24804636

[52] Vollmer W, Joris B, Charlier P, Foster S. 2008. Bacterial peptidoglycan (murein) hydrolases. FEMS Microbiol Rev 32:259–286. doi:10.1111/j.1574-6976.2007.00099.x 18266855

[53] Kohler PL, Hamilton HL, Cloud-Hansen K, Dillard JP. 2007. AtlA functions as a peptidoglycan lytic transglycosylase in the Neisseria gonorrhoeae type IV secretion system. J Bacteriol 189:5421–5428. doi:10.1128/JB.00531-07 17526702 PMC1951824

[54] Sung CK, Li H, Claverys JP, Morrison DA. 2001. An rpsL cassette, janus, for gene replacement through negative selection in Streptococcus pneumoniae. Appl Environ Microbiol 67:5190–5196. doi:10.1128/AEM.67.11.5190-5196.2001 11679344 PMC93289

[55] Higuchi R, Krummel B, Saiki RK. 1988. A general method of in vitro preparation and specific mutagenesis of DNA fragments: study of protein and DNA interactions. Nucleic Acids Res 16:7351–7367. doi:10.1093/nar/16.15.7351 3045756 PMC338413

[56] Miller JH. 1972. Experiments in molecular genetics. Cold Spring Harbor Laboratory, Cold Spring Harbor, N.Y.

[57] Lacks S, Hotchkiss RD. 1960. A study of the genetic material determining an enzyme in Pneumococcus. Biochim Biophys Acta 39:508–518. doi:10.1016/0006-3002(60)90205-5 14413322

[58] Ducret A, Quardokus EM, Brun YV. 2016. MicrobeJ, a tool for high throughput bacterial cell detection and quantitative analysis. Nat Microbiol 1:16077. doi:10.1038/nmicrobiol.2016.77 27572972 PMC5010025

