# Supplementary material for: LytF contributes to pilus extrusion during natural competence in Streptococcus sanguinis SK36
Source: J Bacteriol. 2026 Apr 22;208(5):e00118-26. doi: 10.1128/jb.00118-26 (PMC13192262; doi:10.1128/jb.00118-26)
Supplement: Supplemental Material — Tables S1 to S3; Fig. S1 to S7. [file jb.00118-26-s0001.docx]

**Supplemental Material**

**LytF contributes to pilus extrusion during natural competence in *Streptococcus sanguinis* SK36.**

Rebekka Moe, Katarzyna Wiaroslawa Piechowiak, Leiv Sigve Håvarstein, Morten Kjos and Daniel Straume

Faculty of Chemistry, Biotechnology and Food Science, Norwegian University of Life Sciences, Ås, Norway

**Supplemental tables**

**Table S1.** Bacterial strains used in this study.

| **Strain** | **Relevant characteristics** | **Source or reference** |
| --- | --- | --- |
| *S. pneumoniae* strains | | |
| R704 | R6 derivative, *comA*::*ermAM* | J. P. Claverys^a^ |
| RH425 | R704, but streptomycin resistant | (1) |
| RH426 | RH425, contains the janus cassette downstream of *amiF* | (1) |
| RH2 | R704 having *lacZ* integrated into *hirL* | (2) |
| RM17 | RH425, but Δ*cbpD* | This study |
| MG12 | Clinical strain from NIPH, Δ*cps*::*aad9* |  |
| RM30 | MG12, but Δ*cbpD* | This study |
| *S. sanguinis* SK36 strains | | |
| KP36 | ATCC BAA-1455, but streptomycin resistant | This study |
| KP52 | Δ*comC* |  |
| KP55 | Δ*comC*, Δ*SSA_RS01125* |  |
| KP61 | Δ*comC*, Δ*lytF* |  |
| KP66 | Δ*comC*, Δ*lytF*, Δ*SSA_RS01125* |  |
| DS921 | Δ*comC*, *lacZ*+ |  |
| DS922 | Δ*comC*, Δ*SSA_RS01125*, *lacZ*+ |  |
| DS926 | Δ*comC*, P3-*SSA_RS0112*5, *lacZ+* |  |
| DS934 | Δ*comC*, Δ*SSA_RS01125*, Δ*lytF*, *lacZ*+ |  |
| DS937 | Δ*comE*, *lacZ*+ |  |
| DS939 | Δ*comC*, Δ*comE*, P3-*SSA_RS0112*5, *lacZ*+ |  |
| RM32 | Δ*comC*, pFD116-P*comGA*-*comGC*-*FLAG* |  |
| RM33 | Δ*comC*, Δ*lytF*, pFD116-P*comGA*-*comGC*-*FLAG* |  |
| RM88 | Δ*comC*, Δ*lytF-CHAP*::*lytF-sfgfp* |  |
| RM104 | Δ*comC*, Δ*lytF*::6x*his-lytF* |  |
| RM125 | Δ*comC*, Δ*lytF*, ΔSSA_RS05615::*lytF*-*ermB*, pFD116-P*comGA*-*comGC*-*FLAG* |  |
| RM133 | Δ*comC*, Δ*lytF::lytF_C549A_* |  |
| RM139 | Δ*comC,* Δ*comX*::*comX*-*sfGFP* |  |
| RM140 | Δ*comC,* Δ*lytF*, Δ*comX*::*comX*-*sfGFP* |  |
| *E. coli* strains | | |
| RM18 | DH5α containing pFD116-P*comG*-*comGC*-*FLAG* | This study |

^a^ Gift from J.P. Claverys

**Table S2.** Primers used in this study.

| **Primer** | **Sequence (5’-3’)** | **Application** | **Source** |
| --- | --- | --- | --- |
| Kan484F | GTTTGATTTTTAATGGATAATGTG | Janus cassette | (2) |
| RpsL41R | CTTTCCTTATGCTTTTGGAC |  |  |
| kp171 | ATCAGTATTAGTTCCTGACTCG | Δ*SSA_RS01125*::Janus::DEL | This study |
| kp172 | TGAACCTCCAATAATAAATATTCTCTCCATTCTTCTCTTATC |  |  |
| kp173 | TTTCTAATATGTAACTCTTCCCAATAAAGTGTTTTGTCAAA  TAGAAGAAT |  |  |
| kp174 | CCTGAGTCTCAGGATTGACC |  |  |
| kp175 | GATAAGAGAAGAATGGAGAGAATATTTATTATTGGAGGTTCA |  |  |
| kp176 | ATTCTTCTATTTGACAAAACACTTTATTGGGAAGAGTTACAT  ATTAGAAA |  |  |
| kp177 | GATAAGAGAAGAATGGAGAGAATAAAGTGTTTTGTCAAATA  GAAGAAT |  |  |
| kp178 | ATTCTTCTATTTGACAAAACACTTTATTCTCTCCATTCTTCTCT  TATC |  |  |
| kp179 | ACTGGACTATTCCATCGC | Δ*lytF*::  Janus::DEL |  |
| kp180 | GCCATCCAGGAACTCCTCG |  |  |
| kp181 | GTCCAAAAGCATAAGGAAAGTTTCTAATATGTAACTCTTCCC  AATAAAACTCCTTTGTGAGAATGG |  |  |
| kp182 | CACATTATCCATTAAAAATCAAACTGAACCTCCAATAATAAA  TGTCGCTTTCGCACTTCTC |  |  |
| kp183 | GAGAAGTGCGAAAGCGACAAAACTCCTTTGTGAGAATGG |  |  |
| kp184 | CCATTCTCACAAAGGAGTTTTGTCGCTTTCGCACTTCTC |  |  |
| kp193 | CGACCTTATTAGCGGTATACC | *lytF* |  |
| kp194 | GACCTTCAGTGGTATGACG |  |  |
| kp195 | TGGCTATGTTGACTCAGACG | *SSA_RS01125* |  |
| kp196 | AATCATATTTCTGAGAATATGGG |  |  |
| kp207 | TTATATGAAGTAGTTACACTGG | Δ*comC*::Janus::DEL |  |
| kp208 | TGAACCTCCAATAATAAATAACTATCTCCTATCTTTTTATCTTG |  |  |
| kp209 | TTTCTAATATGTAACTCTTCCCAATGAATTTTAAAGTTTAGGG  GAGA |  |  |
| kp210 | AGCTGAATCTGATGTTCACG |  |  |
| kp211 | CAAGATAAAAAGATAGGAGATAGTTATTTATTATTGGAGGTT  CA |  |  |
| kp212 | TCTCCCCTAAACTTTAAAATTCATTGGGAAGAGTTACATATT  AGAAA |  |  |
| kp213 | CTATTCTTTTCAAATTGCTTTAAATAGC |  |  |
| kp214 | GCTATTTAAAGCAATTTGAAAAGAATAG |  |  |
| kp215 | ATTCGCCTTCTAAGCGAACG |  |  |
| kp216 | CGGAACTATAATCTCAAGACC |  |  |
| kp217 | AATCCAATTATTTCAAGTGACATAACTATCTCCTATCTTTTTA  TCTTG |  |  |
| kp218 | CAAGATAAAAAGATAGGAGATAGTTATGTCACTTGAAATAA  TTGGATT |  |  |
| kp219 | CATGATGGATACTACAGTGC |  |  |
| kp220 | CTACTAATCATCAGAATCTGAG |  |  |
| ds703 | TGTCCAAGCTGGTCAAGGAAC | Δ*hirL*::Janus |  |
| ds704 | CACATTATCCATTAAAAATCAAACGTTCAAGCCTCCTTGATT  CAC |  |  |
| ds705 | GTCCAAAAGCATAAGGAAAGAGAAAATTCAGAATTATTTAA  TTTGTTC |  |  |
| ds706 | TCCTGGACGAGAATTACGAC |  |  |
| ds711 | TTGCACTGTCCCCCTGGTATAATAACTATACATGCAAGATCTA  AATAGGAGGAAAATTAGTGGAAGTTACTGACGTAAGATTAC | Δ*hirL*::Janus::P3-*lacZ* |  |
| ds712 | TTATTTTTGACACCAGACCAAC |  |  |
| ds713 | TACCAGGGGGACAGTGCAAGTTCAAGCCTCCTTGATTCAC |  |  |
| ds714 | GTTGGTCTGGTGTCAAAAATAAAGAAAATTCAGAATTATT  TAATTTGTTC |  |  |
| ds720 | GGCTTGTTTCTGACATGTATC | Δ*comE*::Janus |  |
| ds721 | CACATTATCCATTAAAAATCAAACAAATTCTATCTCCTAATTG  TTAAAATC |  |  |
| ds722 | GTCCAAAAGCATAAGGAAAGAAACCTGATATAATGGAATATG  TTC |  |  |
| ds723 | AAGTAGATGCTATAACTACTAATG |  |  |
| rm032 | GATCACTAGTAAACTAAAAACCACTAAGCCTCTTT | pFD116-P*comG*-*comGC*-*FLAG* |  |
| rm033 | ATGAAAAAATTTAATACCTTAAAAGTTCA |  |  |
| rm034 | CATGCCATGGTTACTTGTCGTCATCGTCTTTGTAGTCATTGGCAACCGCTTGA |  |  |
| rm035 | TGAACTTTTAAGGTATTAAATTTTTTCATATATCCTCCTCACCTTACTATTCG |  |  |
| rm120 | TGTATTGGGTCCCTTGTCGC | *lytF-CHAP*::*lytF-sfGFP* |  |
| rm121 | CACCTTTAGAACCGGTAAGATGTTTACCACCACCACCACCCTCAACCTGATAGCTAGTTCCAGTC |  |  |
| rm122 | GACTGGAACTAGCTATCAGGTTGAGGGTGGTGGTGGTGGTAAACATCTTACCGGTTCTAAAGGTG |  |  |
| rm123 | GAGAAGTGCGAAAGCGACTTATTATGCGGCCGCTCCACTAG |  |  |
| rm124 | CTAGTGGAGCGGCCGCATAATAAGTCGCTTTCGCACTTCTC |  |  |
| rm125 | ATCAGACGGCCTTGCCACTG |  |  |
| rm129 | CACATTATCCATTAAAAATCAAACAAAACTCCTTTGTGAGAATGGTTTTGG |  |  |
| rm130 | CCAAAACCATTCTCACAAAGGAGTTTTGTTTGATTTTTAATGGATAATGTG |  |  |
| rm131 | CGTCCAAAAGCATAAGGAAAGGTCGCTTTCGCACTTCTCAAAACTAC |  |  |
| rm132 | GTAGTTTTGAGAAGTGCGAAAGCGACCTTTCCTTATGCTTTTGGACG |  |  |
| rm152 | TTGAGAAGTGCGAAAGCGACTTATTTAGGGTAGATATAGGTCAGGC | *6x-his*-*lytF* |  |
| rm153 | GTCGCTTTCGCACTTCTCAA |  |  |
| rm154 | ATGATGATGATGATGATGCGCATGGACTGTTGAGTGG |  |  |
| rm155 | CATCATCATCATCATCATACCGAAACGCCTGAATCTG |  |  |
| ds121 | TTATAATTTTTTTAATCTGTTATTTAAATAG | Δ*S*SA_RS01125::*aad9* |  |
| ds717 | TTAAATGTGCTATAATACTAGAAAATACTTGTGGAGGTTCCATTGTGAGGAGGATATATTTGAATAC |  |  |
| rm161 | CTATTTAAATAACAGATTAAAAAAATTATAAAAAGTGTTTTGTCAAATAGAAGAATTATGG |  |  |
| rm162 | TCCACAAGTATTTTCTAGTATTATAGCACATTTAAATTCTCTCCATTCTTCTCTTATCATTATAGAAGAAG |  |  |
| rm203 | ACTCAAGGATACAACGATTGTCTCAGC | ΔSSA_RS05615::*lytF*-*ermB* |  |
| rm204 | ACGTCCAAAAGCATAAGGAAAGTTAAAAACACCCCAAAAGTTAGATTTTTTCTG |  |  |
| rm205 | GATTATATCACATTATCCATTAAAAATCAAACTTATTTGTCTAACTTTTTGGGGTCAGTACAC |  |  |
| rm206 | GCTCAGGCCCTGCTAAGTCG |  |  |
| rm207 | TTATTTAGGGTAGATATAGGTCAGGCG |  |  |
| rm208 | TCCATTCTAATGAGAAAAAGAATATCTCTTCTATATGATCG |  |  |
| rm209 | CGCCTGACCTATATCTACCCTAAATAATTAAAAACACCCCAAAAGTTAGATTTTTTCTG |  |  |
| rm210 | ATCATATAGAAGAGATATTCTTTTTCTCATTAGAATGGATTATTTGTCTAACTTTTTGGGGTCAGTACAC |  |  |
| rm270 | GGTCAAGCTACTTGGGGAGC | Δ*lytF*::*lytF*_C549A_ |  |
| rm271 | CTCCCCAAGTAGCTTGACCG |  |  |
| rm286 | GGACATACTATTGTTGGTTTGGTATTATC | Δ*comX*::*comX*-Janus |  |
| rm287 | CACATTATCCATTAAAAATCAAACTTACAAAATCGTATTATCTTGAAAATCTTTTAAGTG |  |  |
| rm288 | CGTCCAAAAGCATAAGGAAAGTGAACAAACTTGATGACTTTATTTCTG |  |  |
| rm289 | CCATATATCTACGCATTTCACCG |  |  |
| rm290 | CCTCCTTATTAGTTAATCAGTATCTAGTGGATCTTACAAAATCGTATTATCTTGAAAATCTTTTAAGTG | Δ*comX*::*comX*-*sfGFP* |  |
| rm291 | GATCCACTAGATACTGATTAACTAATAAGGAGG |  |  |
| rm292 | GGCATGGATGAGCTTTATAAGTAATGAACAAACTTGATGACTTTATTTCTG |  |  |

**Table S3.** Transformation efficiency of *S. sanguinis* mutants at different ODs during exponential growth phase.

|  | KP52 (Δ*comC*) | KP61(Δ*comC*, Δ*lytF*) | KP55 (Δ*comC*, Δ*S*SA_RS01125) | KP66 (Δ*comC*, Δ*S*SA_RS01125, Δ*lytF*) |
| --- | --- | --- | --- | --- |
| OD_550_ | Transformation  efficiency (%)* | Transformation  efficiency (%) | Transformation  efficiency (%) | Transformation  efficiency (%) |
| 0.05 | 0.007 | 0.003 | 0.017 | 0.0016 |
| 0.1 | 0.018 | 0.002 | 0.01 | 0.0007 |
| 0.2 | 0.04 | 0.0014 | 0.048 | 0.0022 |
| 0.3 | 0.036 | 0.0035 | 0.029 | 0.0017 |
| 0.4 | 0.054 | 0.0033 | 0.055 | 0.0024 |
| 0.5 | 0.012 | 0.0011 | 0.011 | 0.0008 |
| 0.6 | 0.002 | 0.0001 | 0.0033 | 0.0001 |
| 0.7 | 0.0001 | 0 | 0 | 0 |
| 0.8 | 0 | 0 | 0 | 0 |
| 0.9 | 0 | 0 | 0 | 0 |

* Calculated as the number of CFUs of transformants/total number of CFUs in the culture.

**Supplemental figures**


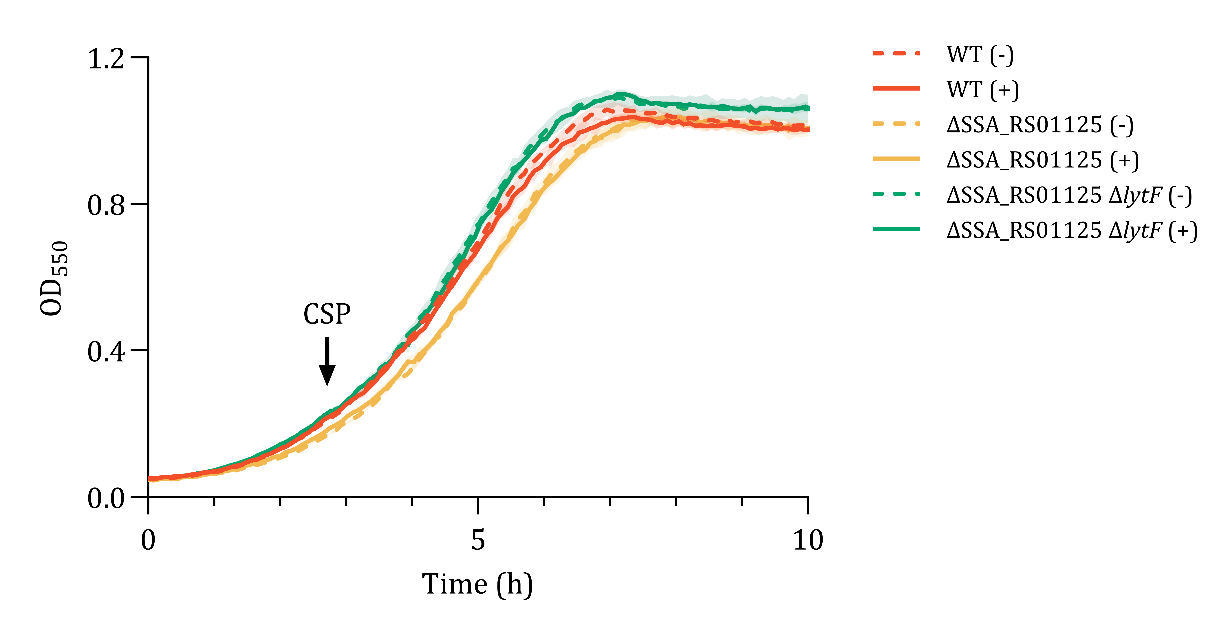


**Fig. S1** Growth curve assay of competent (+) and non-competent (-) *S. sanguinis* WT (KP52), Δ*SSA_RS01125* (KP55), and Δ*SSA_RS01125*, Δ*lytF* (kP66). At OD_550_ = 0.2, the (+)-cultures were induced to competence by the addition of CSP (final conc. 250 ng/mL) and the (-)-cultures were supplied with the same volume of PBS. Results are based on data from three biologically independent experiments.


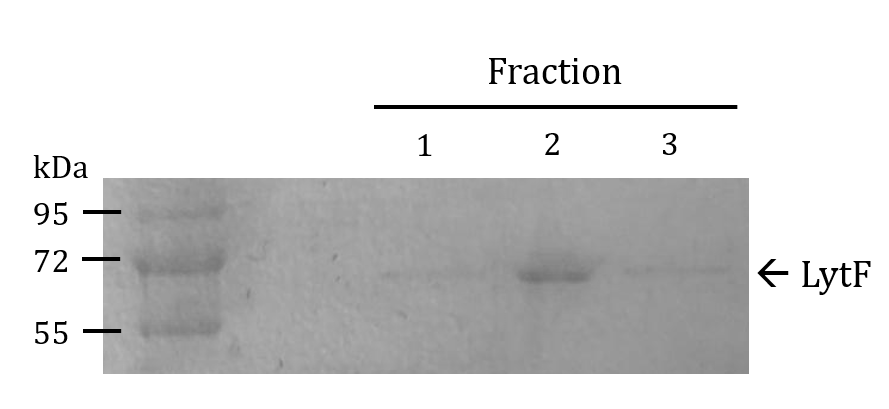


**Fig. S2** SDS-PAGE of immobilised metal affinity chromatography (IMAC) purified His_6_-LytF. The histidine-encoding sequence was inserted just downstream of the secretion signal sequence in *lytF*. The protein was expressed natively and purified form the supernatant of the competent culture. The numbers 1, 2, and 3 correspond to elution fractions with the highest A280 nm absorbance during imidazole elution from the Ni-NTA column. A protein band corresponding to the size of His_6_-LytF (73 kDa) was visualised by Coomassie brilliant blue staining.


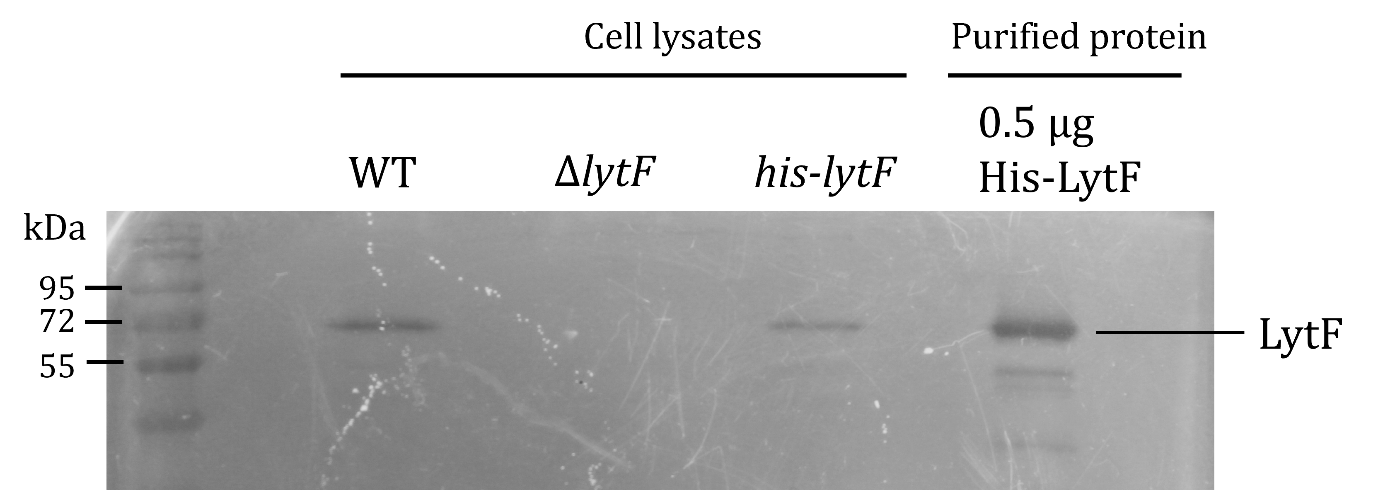


**Fig. S3** Zymogram of cell lysates of *S. sanguinis* WT (KP52), Δ*lytF* (KP61), and *his-lytF* (RM104), and purified His-LytF. Cells of KP52 were incorporated into the gel as the substrate. Cell lysate samples were prepared from competence-induced cultures. A total of 0.5 of the purified His_6_-LytF protein was loaded onto the gel. The clear bands corresponding to the size of His_6_-LytF (73 kDa) show murealytic activity of LytF against the substrate strain. The amount loaded corresponds to 350 µL of the original 10 mL culture, meaning the amount of LytF produced natively in a culture of this volume is considerably lower than 0.5 µg.


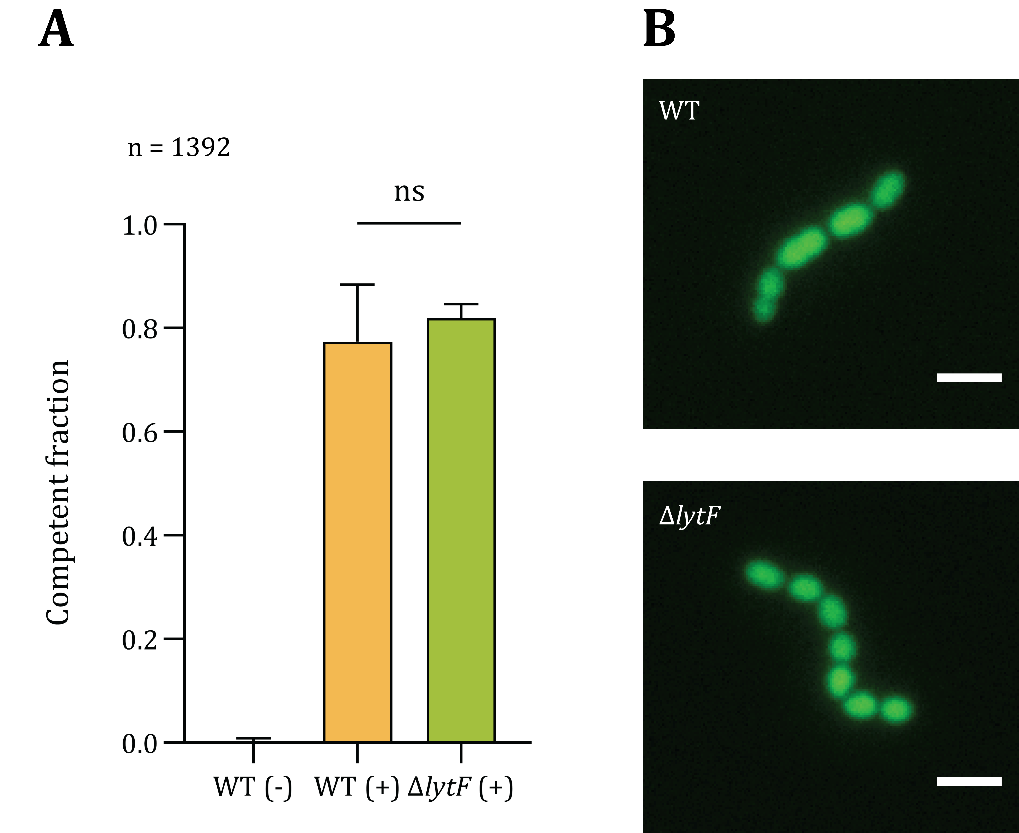


**Fig. S4** Fraction of competent cells in cultures of wild-type (RM139) and Δ*lytF* (RM140) with a *sfGFP*-reporter downstream of *comX*. Panel A: Fraction of competent cells (expressing *comX*-*sfGFP*) measured from microscopy images of induced (+) and non-induced (-) cultures using the MicrobeJ plugin in ImageJ (3, 4). The difference in means was determined to be statistically non-significant using a Student’s t-test (p > 0.05). Total number of cells counted: 1,392. Panel B: Microscopy images of induced wild-type-like (RM139) and Δ*lytF* (RM140). Scale bars are 2 µM.


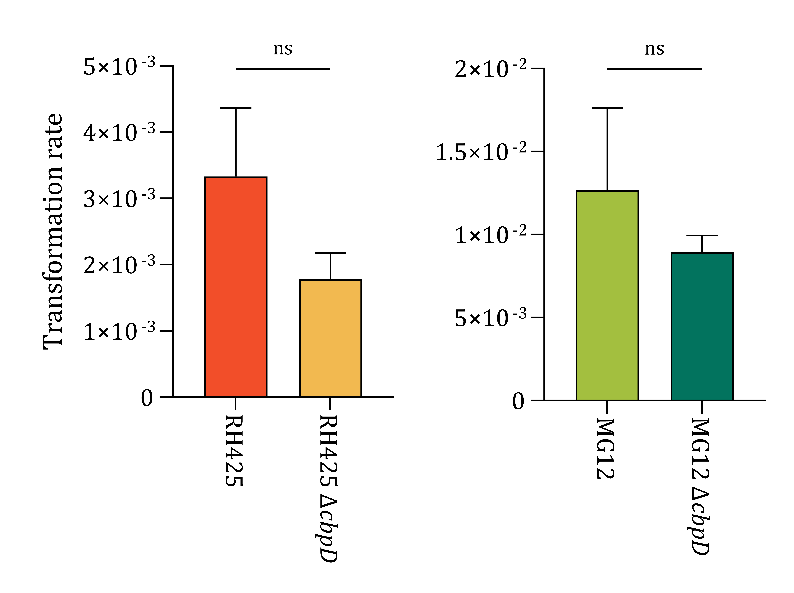


**Fig. S5** Transformation rate of *S. pneumoniae* Δ*cbpD*-mutants of a lab strain (RH425) and a clinical isolate (MG12) compared to their parental strains. The transformation rate was calculated as the number of CFUs of transformants divided bythe total number of CFUs in the culture. The difference in transformation rates between the Δ*cbpD*-mutant and the parental strain was non-significant (Student’s t-test, p = 0.2666). Results are based on data from three biologically independent experiments.


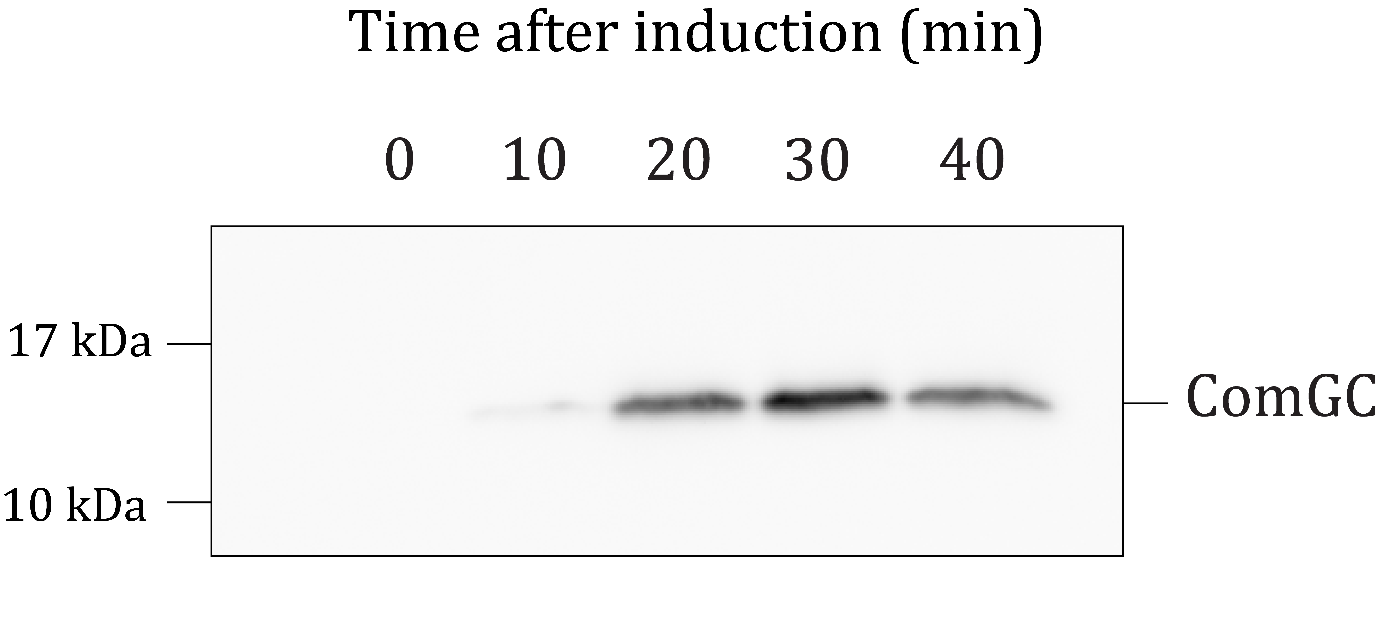


**Fig. S6** Time-series immunoblot of ComGC-FLAG to assess peak expression. Competence was induced in a 10 mL culture at OD_550_ = 0.2 and incubated for 10 to 40 minutes with increments of 10. A non-induced culture was included as a negative control (time = 0). ComGC-FLAG in samples of lysed cell extracts were detected using anti-FLAG antibodies. The strongest signal was detected 30 minutes after competence induction.


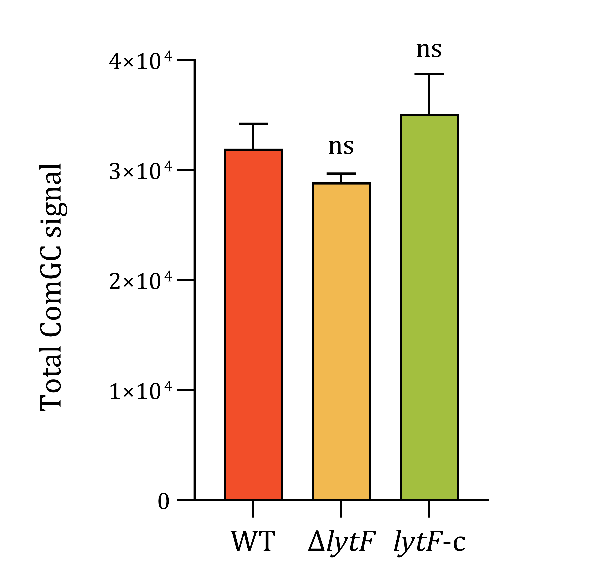


**Fig. S7** Effect of LytF on total levels of ComGC-FLAG of the wild-type (RM32), a *lytF*-knockout (RM33), and a *lytF*-complementation strain (RM125). The ComGC-FLAG signal intensities from immunoblots of extracellular and intracellular fractions were measured using ImageJ (5) and summarised to estimate the total relative ComGC-FLAG amount. The difference in means between the strains was determined to be statistically non-significant using a One-way ANOVA and Dunnett’s test (p > 0.05). Results are based on data from three biologically independent experiments.

**References**

1. Johnsborg O, Håvarstein LS. 2009. Pneumococcal LytR, a protein from the LytR-CpsA-Psr family, is essential for normal septum formation in Streptococcus pneumoniae. J Bacteriol 191:5859–64.

2. Johnsborg O, Eldholm V, Bjørnstad ML, Håvarstein LS. 2008. A predatory mechanism dramatically increases the efficiency of lateral gene transfer in Streptococcus pneumoniae and related commensal species. Molecular Microbiology 69:245–253.

3. Ducret A, Quardokus EM, Brun YV. 2016. MicrobeJ, a tool for high throughput bacterial cell detection and quantitative analysis. Nature Microbiology 1:1–7.

4. Rueden CT, Schindelin J, Hiner MC, DeZonia BE, Walter AE, Arena ET, Eliceiri KW. 2017. ImageJ2: ImageJ for the next generation of scientific image data. BMC Bioinformatics 18:529.

5. Schneider CA, Rasband WS, Eliceiri KW. 2012. NIH Image to ImageJ: 25 years of image analysis. Nat Methods 9:671–5.
